# A metamorphic testing approach for event sequences

**DOI:** 10.1371/journal.pone.0212476

**Published:** 2019-02-19

**Authors:** Jing Chen, Yinglong Wang, Ying Guo, Mingyue Jiang

**Affiliations:** 1 College of Computer Science and Engineering, Shandong University of Science and Technology, Qingdao, Shandong, China; 2 Shandong Provincial Key Laboratory of Computer Networks, Shandong Computer Science Center (National Supercomputer Center in Jinan), Qilu University of Technology (Shandong Academy of Sciences), Jinan, Shandong, China; 3 School of Information Science, Zhejiang Sci-Tech University, Hangzhou, Zhejiang, China; Fraunhofer USA, UNITED STATES

## Abstract

Test oracles are commonly used in software testing to determine the correctness of the execution results of test cases. However, the testing of many software systems faces the test oracle problem: a test oracle may not always be available, or it may be available but too expensive to apply. One such software system is a system involving abundant business processes. This paper focuses on the testing of business-process-based software systems and proposes a metamorphic testing approach for event sequences, called *MTES*, to alleviate the oracle problem. We utilized event sequences to represent business processes and then applied the technique of metamorphic testing to test the system without using test oracles. To apply metamorphic testing, we studied the general rules for identifying metamorphic relations for business processes and further demonstrated specific metamorphic relations for individual case studies. Three case studies were conducted to evaluate the effectiveness of our approach. The experimental results show that our approach is feasible and effective in testing the applications with rich business processes. In addition, this paper summarizes the experimental findings and proposes guidelines for selecting good metamorphic relations for business processes.

## Introduction

Software is widely used in various fields and greatly promotes the development of society. However, software faults have caused massive disasters. Software quality assurance has become a critical activity in the software industry, and software testing is an effective method to ensure software quality. Many techniques have been proposed to guide test case selection and testing automation to improve the effectiveness of software testing. Most of these techniques require an underlying assumption that an oracle (a mechanism through which testers can verify the correctness of the test outputs) is attainable. However, in many practical applications, a test oracle is not attainable or is attainable but is too expensive to apply. These two situations are known as the oracle problem [1–3] and are challenging problems in software testing.

In real-life applications, a system often consists of many subsystems or services that involve a large number of business processes and data transformations. Such a system is very difficult to test. Testers not only need to identify business processes and construct many test inputs but also have to determine the expected outputs. This process is error-prone and expensive. For example, a bank system normally involves many complex transaction processes from various terminals and frequently processes transactions in batches. To test such a system thoroughly, testers have to identify a large number of business processes, construct a large number of test cases and calculate the expected outputs manually. The expectations and comparisons of the test outputs are time-consuming and error-prone. Therefore, test oracles are expensive to apply and the testing of such software systems faces the oracle problem.

Traditionally, one way to test a system that suffers from the test oracle problem is to use a ‘pseudo-oracle’ [4], in which multiple implementations of an algorithm are executed and at least one fault is detected if the outputs are different. This method is not always feasible because it is very costly, and different people can make the same type of mistake. Another method is a ‘partial oracle’ [5], which can verify the correctness or incorrectness of test outputs according to a certain condition or range. For instance, the output of sin 38° should not be greater than 1 or less than −1. This method is relatively simple and inexpensive, but it is suitable only for limited cases. Metamorphic testing (MT) has been proposed to alleviate the oracle problem [6, 7]. To address the oracle problem, MT uses the relations over multiple inputs and outputs, namely, metamorphic relations (MRs), to verify the test results. If an MR is violated, at least one fault is detected. MT is a simple, effective and automatable method without test oracles [8, 9]. Many researchers have applied MT in various applications in different domains, such as numerical analysis [10], machine learning [11], bioinformatics [12, 13], middleware applications [14], embedded software [15], the National Aeronautics and Space Administration (NASA) data access toolkit [16], cybersecurity [17], compilers [18, 19], search engines [20] and geographic systems [21]. Additionally, MT has also been integrated with other testing and analysis techniques, such as fault-based testing [7], program slicing [22] and symbolic execution [23]. A comprehensive survey of MT introduces its application areas, research results and challenges [24].

In the software industry, a system usually includes a large number of interactions and business processes. End-users pay more attention to the correctness of business processes. The application of MT to test business processes is challenging. Two prominent problems exist. One problem is how to represent test inputs in MT for business processes. To test the system thoroughly, testers need to construct various test scenarios from the users’ perspectives to reflect business processes. These test scenarios should also be regarded as test inputs in the testing of business-process-based systems. Test scenarios are basically expressed in natural language. How to express the relations between different test scenarios in MT must be studied. One possible approach is to formalize test scenarios just like normal test inputs in MT for business processes. Another problem is how to construct an MR for business processes. The MR requires multiple relations between different test scenarios, composite test inputs and outputs, among which the key challenge is to construct the relations among different test scenarios.

Some previous studies regarding event sequence testing can help us solve the first problem. Belli et al. proposed event sequence graphs (ESGs) to represent a user’s actions in graphical user interface (GUI) testing [25]. Memon proposed a scalable event-flow model of GUI-based applications to present all possible event sequences on a GUI [26]. Sabharwall et al. proposed an event-flow model to generate and express test scenarios [27]. A sequence generation approach to business process testing was proposed based on test case composition and colored petri nets [28]. In addition, solutions to event sequence testing, such as sequence covering arrays [29], better bounds [30], integrating event-based testing and structure testing [31], have been proposed. Clearly, using event sequences is an intuitive approach to test business-process-based systems. The abovementioned methods of event sequence generation provide guidance regarding the formal description of test scenarios and facilitate the descriptions of the test input and MRs. The foremost step of MT for event sequences is to construct useful MRs between event sequences. Although previous studies presented some principles for constructing good MRs in MT (see the section on related work), they did not include how to construct MRs between event sequences.

This paper proposes an MT approach for event sequences. We utilize event sequences to represent business processes and then construct MRs for event sequences to test business-process-based software systems without using test oracles. To apply this method, we study general rules that we call ‘properties between event sequences’ to identify MRs for event sequences. Three case studies are conducted to demonstrate the specific MRs. The experimental results and findings demonstrate the effectiveness of our approach.

## Background

### Metamorphic testing

MT can be used to test systems with or without test oracles [32]. Instead of focusing on the verification of the correctness of each individual output, MT identifies various MRs to verify the relations among multiple inputs and their outputs. In general, one or more MRs are first identified based on knowledge about the intended algorithm or functionality of the software under test (SUT). Then, the source test cases are generated using traditional testing techniques, such as random testing [33], fault-based testing [7], black-box testing and white-box testing. Given a source test case, its follow-up test case is constructed by using the relevant MR. These source and follow-up test cases are further executed on the SUT, and their outputs are checked against the MR. If the MR is violated, then the SUT must be faulty.

A simple example that exemplifies MT is a program *P* that calculates the median of a set of numbers. The correctness of *P* is difficult to verify when the number of elements in the set is large. However, the algorithm of *P* has some defined properties. One property is that when every input number is increased by the same real number *x*, the resulting median is also increased by *x*. Based on this property, we can define an MR as follows “Suppose the source test input is {*s*_1_, *s*_2_, .., *s*_*n*_} (*n* is the number of input elements and *n* >= 1), and the follow-up test case is constructed as {*s*_1_ + 10, *s*_2_ + 10, …, *s*_*n*_ + 10} based on the source test case. Then, we have *P*(*s*_1_ + 10, *s*_2_ + 10, …, *s*_*n*_ + 10) = *P*(*s*_1_, *s*_2_, …, *s*_*n*_) + 10.” Then, the source and follow-up test cases are both executed in the program *P*, and their outputs are compared. If this MR is violated, there exists at least one fault in the program.

MT provides an effective verification mechanism of test outputs for applications with the oracle problem. Rather than verifying the individual output of one execution, MT determines whether an MR is violated on the basis of multiple executions. The method is simple to implement and independent of the programming language. Additionally, the automation of MT is easy. We can write simple scripts to automatically generate follow-up test cases and compare test outputs. MT has been applied in a wide range of applications. Although some general rules have been proposed to select good MRs, how to identify an MR for business processes has rarely been studied. We study this issue in this paper.

### Business process and event sequence graph

**Fig 1:**
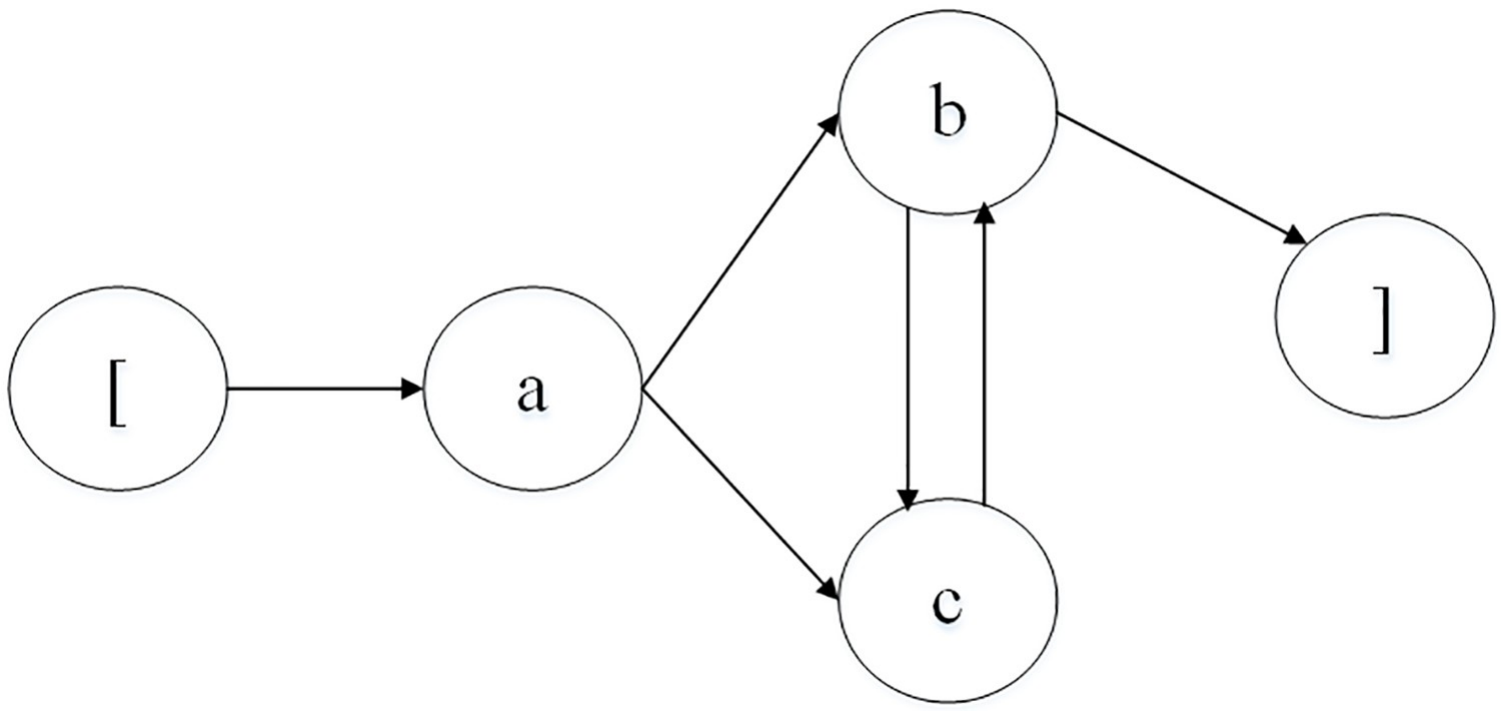
An ESG with pseudo entry and exit nodes.

A business process is a series of activities performed in a coordinated manner to achieve a business goal [34]. A business process can be described as an ESG, which is a directed graph that depicts events and event interactions in a simplified way [25]. An illustrative example of an ESG is shown in Fig 1. A node denotes an event, which indicates a user’s action or an operation call with inputs to the SUT. An arrowed line represents the interaction between two events. Two pseudo-nodes ‘[’,‘]’ inserted into an ESG do not represent real events but rather mark the entry and exit of the ESG.

A test scenario of a business process depicts a sequence of operations or interactions between a user and a system. The scenario can be described as an event sequence composed of well-organized events. Thus, various event sequences representing test scenarios of business processes can be generated based on an ESG and search methods, such as deep-breath-first search. These sequences can be 1-, 2-, 3-, …*n*-way event sequences and can be tested based on event coverage or basis path testing. To obtain an event coverage of 100%, all events must be performed at least once. Basis path testing is a white-box testing method that finds linearly independent paths of execution in the control flow graph (CFG) to test a program. A linearly independent path, which we call a basis path, is a path through a CFG with at least one node different from the nodes of the other paths. An ESG is similar to the CFG of a program. All linearly independent paths are constructed and executed to cover all branches of event sequences in an ESG. For instance, in Fig 1, event *b* is executed after event *a* is performed, and events *c* and *d* follow event *b*. Thus, event sequence 〈*a*, *b*, *c*, *d*〉 depicts a business process scenario that traverses the path *a* → *b* → *c* → *d*. The path covers all events for an event coverage of 100% but covers only one independent path of execution, i.e., *a* → *b* → *c* → *d*. More event sequences, such as 〈*a*, *c*, *d*〉 and 〈*a*, *b*, *d*〉, should be performed to obtain greater path coverage.

**Fig 2:**
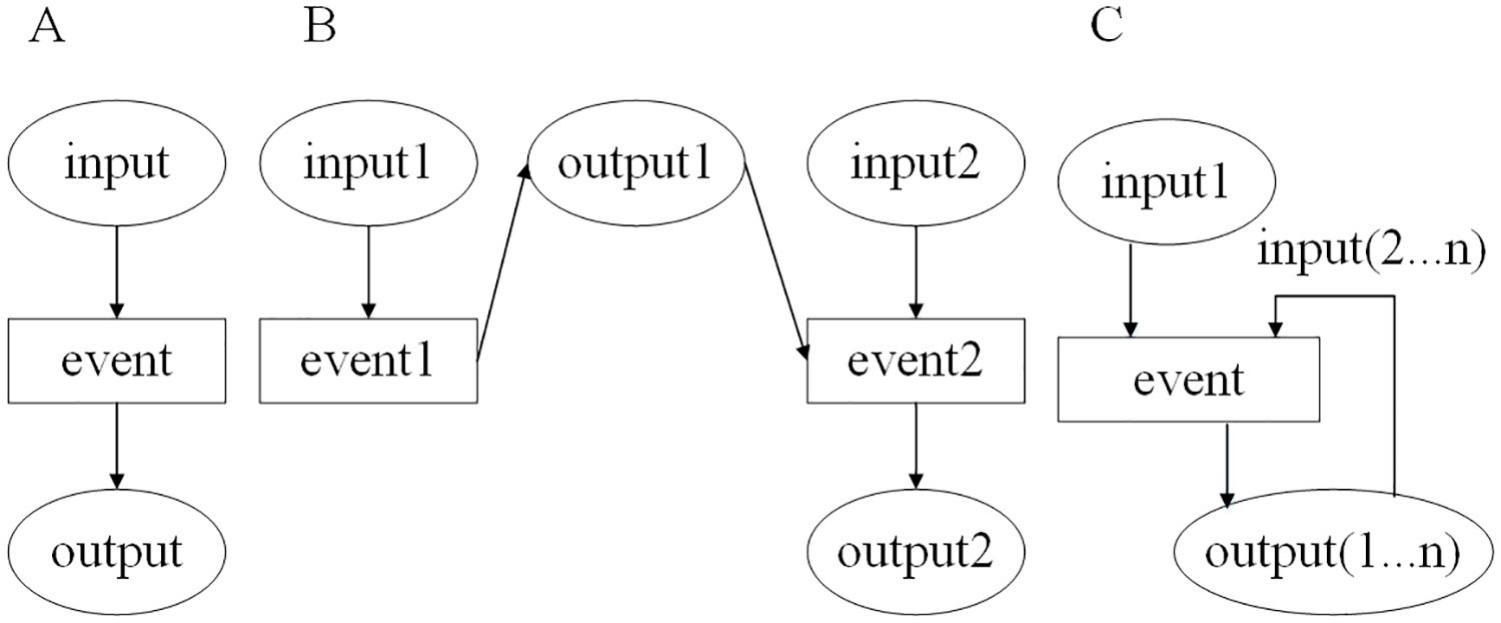
Three basic business process scenarios. A: A scenario of a single event. B: Scenario of a sequential event sequence. C: Scenario of a loop event sequence.

**Fig 3:**
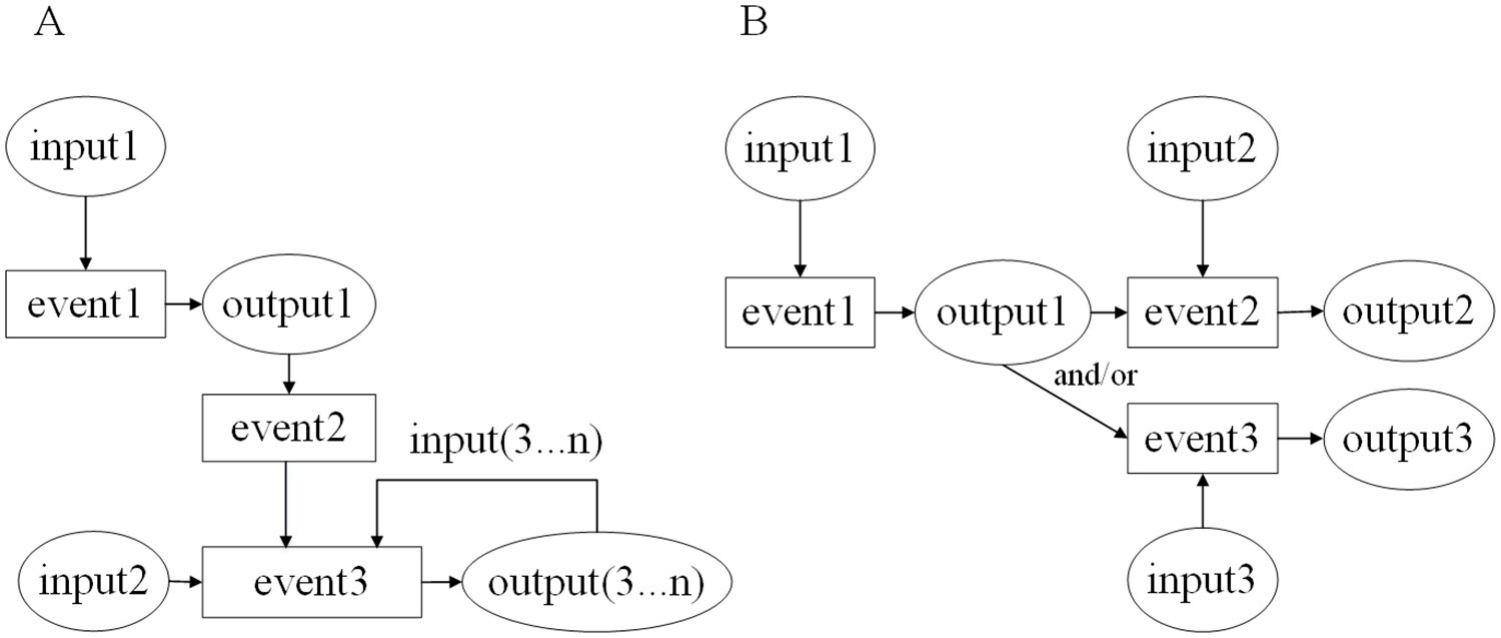
Combination scenarios of basic business processes. A: Combination of a sequential event sequence and a loop event sequence. B: Parallel or alternative event sequence.

The three basic business process scenarios are shown in Fig 2. Fig 2A shows a scenario in which only a single event is tested. In some cases, an event can be tested after another event. These two events can be closely or slightly related. For instance, *event*2 in Fig 2B is executed after *event*1 is performed successfully. This is a typical sequential event sequence. Sometimes, a loop event can be executed many times, and the input of the later event may come from the output of the previous event. Fig 2C describes a loop event sequence. Other scenarios can be combined with the basic scenarios. Fig 3A shows a scenario that combines a sequential event sequence 〈*event*1, *event*2〉 with a loop event sequence 〈*event*3_1_, …, *event*3_*n*_〉 (the subscripts 1, …, *n* represent the number of loop executions). In Fig 3B, the scenario can be divided into two parallel sequential event sequences 〈*event*1, *event*2〉 and 〈*event*1, *event*3〉 when the condition ‘and’ holds. If the condition ‘or’ holds, only one of the event sequences 〈*event*1, *event*2〉 or 〈*event*1, *event*3〉 exists. Thus, MT for business processes can be transformed into metamorphic testing for event sequences.

### Running examples

A spreadsheet is an application created by end-users that displays a table of information for end-users’ tasks, such as data analysis, mathematical computation and office work. A typical spreadsheet may consist of hundreds or even thousands of cells into which input data (e.g., text and numbers) and formulas are entered. Spreadsheets are error-prone for end-users due to mistyping input data and formulas. Moreover, incorrect input data and formula faults can spread from the upstream cells to the downstream cells that depend on the upstream input data or computation results. These errors are difficult to detect. Although oracles are available for spreadsheets, the testing is time-consuming and prone to human error because testers generally must manually calculate the ‘expected’ results. This issue causes the oracle problem in spreadsheet testing, which has been reported in many previous studies [35–37]. The following two examples involve a formula fault and incorrect input data.

Example 1 is a spreadsheet in which cells A2-A301 list the daily sales amounts, and cell A302 uses a faulty formula ‘=SUM(A2:A300)/300’ instead of the correct one ‘=SUM(A2:A301)/300’ to calculate the average daily sales amount. To verify the correctness of this spreadsheet, a tester manually calculates the expected result with a calculator and compares it with the ‘actual’ value in cell A302. This manual computation is time-consuming and prone to error owing to the large amount of data. MT can use some properties to alleviate this problem. An example of an MR is given as follows. We execute two test cases and compare whether their output results satisfy MR1. If this MR is not satisfied, there exists a fault in this spreadsheet.

MR1: If all daily sales amounts in cells A2-A301 increase by a constant *k*, the average daily sales amount in cell A302 will increase by *k*.

**Fig 4:**
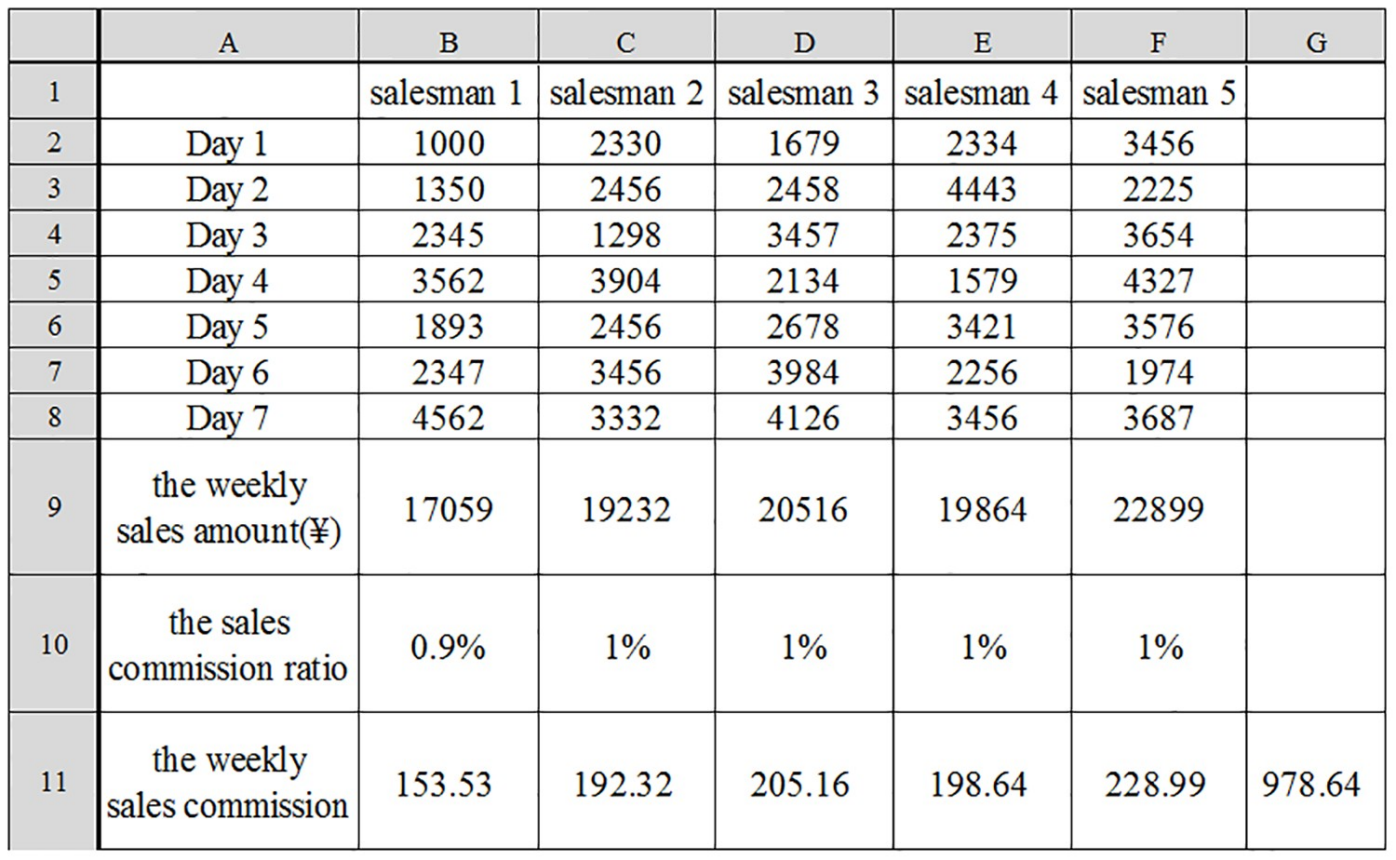
A spreadsheet in example 2.

Example 2 shows a spreadsheet involving multistep computations in Fig 4. Each of the columns from B to F displays one salesman’s data. All salesmen’s daily sales amounts are stored in row 2 to row 8. Each of the cells from B9 to F9 stores each salesman’s weekly sales amount calculated via the summation formula. For instance, the value in cell B9 is calculated by the formula ‘=SUM(B2:B8)’. Cells B10-F10 show the sales commission ratios for all salesmen’s weekly sales amounts. The weekly sales commissions in cells B11-F11 are obtained by multiplying the weekly sales amounts by the sales commission ratios. For example, the value in cell B11 is calculated using the formula ‘=B9 * B10’. Finally, the total of all salesmen’s sales commissions is calculated using the formula ‘=SUM(B11:F11)’ in cell G11. Here, cell B10 stores not the correct sales commission ratio of 1% but rather the wrong input value of 0.9%. The subsequent weekly sales commission in cell B11 is also faulty, which further causes a faulty result in cell G11.

Generally, a tester computes the results in cells B9-F9 manually. Then, these results are manually multiplied by the values of cells B10-F10 to obtain the weekly sales commissions in cells B11-F11. Finally, the values in cells B11-F11 are calculated to generate the expected result in cell G11. From this process, we can see that a tester manually performs mathematical calculations 11 times to obtain the expected result for such a simple spreadsheet. If the spreadsheet includes a large amount of data, it will be even more expensive to obtain the oracle due to the considerable number of error-prone manual computations.

**Fig 5:**
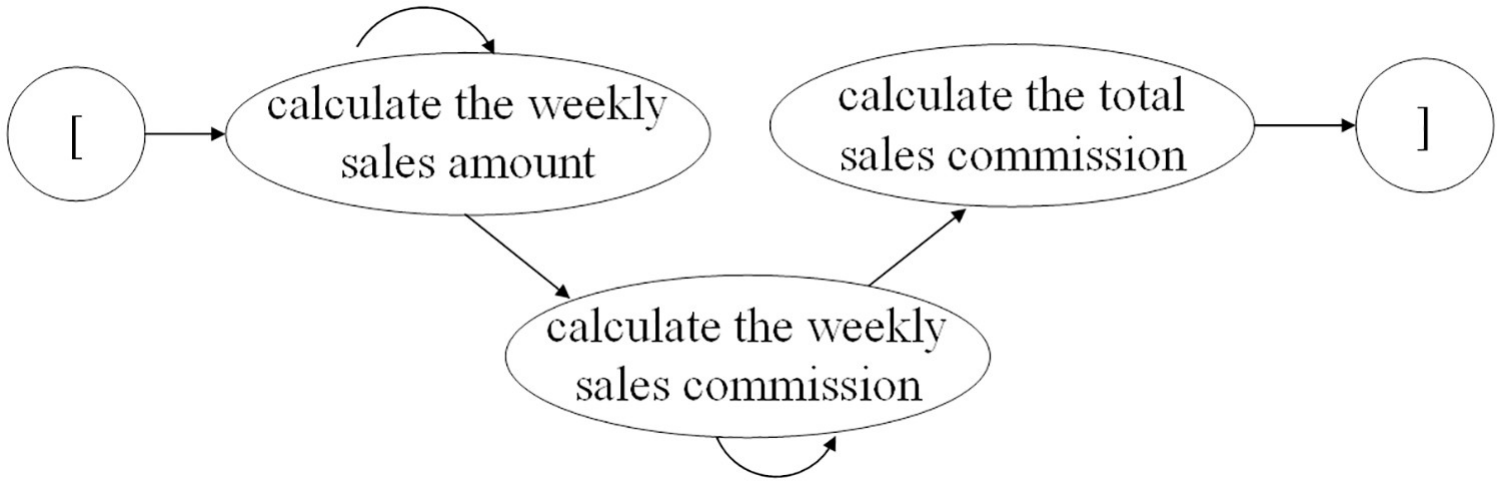
ESG for the process of multistep computations.

MT can simply use an MR to solve the oracle problem in spreadsheet testing. The above process implies three events: calculate the weekly sales amount, calculate the weekly sales commission and calculate the total sales commission. We can use an event sequence to represent the process of the multistep computation and construct the ESG in Fig 5. An example of an MR for this event sequence is as follows.

MR2: For the event sequence ‘calculate the weekly sales amount, calculate the weekly sales commission, calculate the total sales commission’, the total sales commission in cell G11 should increase by the constant 0.01 * *m* if all daily sales amounts in the spreadsheet increase by a constant *m*.

MT for the event sequence can test the spreadsheet more easily by executing only two groups of test data. Certainly, the correctness of the spreadsheet can be further verified in a finer-grained manner, such as by considering each salesman’s weekly sales commission, and the following MR3, an extension of MR2, can be used.

MR3: For the event sequence ‘calculate the weekly sales amount, calculate the weekly sales commission, calculate the total sales commission’, each weekly sales commission in cells B11-F11 and the total sales commission in cell G11 will increase by the constant 0.01 * *m* if all daily sales amounts in the spreadsheet increase by a constant *m*.

## Methods

**Fig 6:**
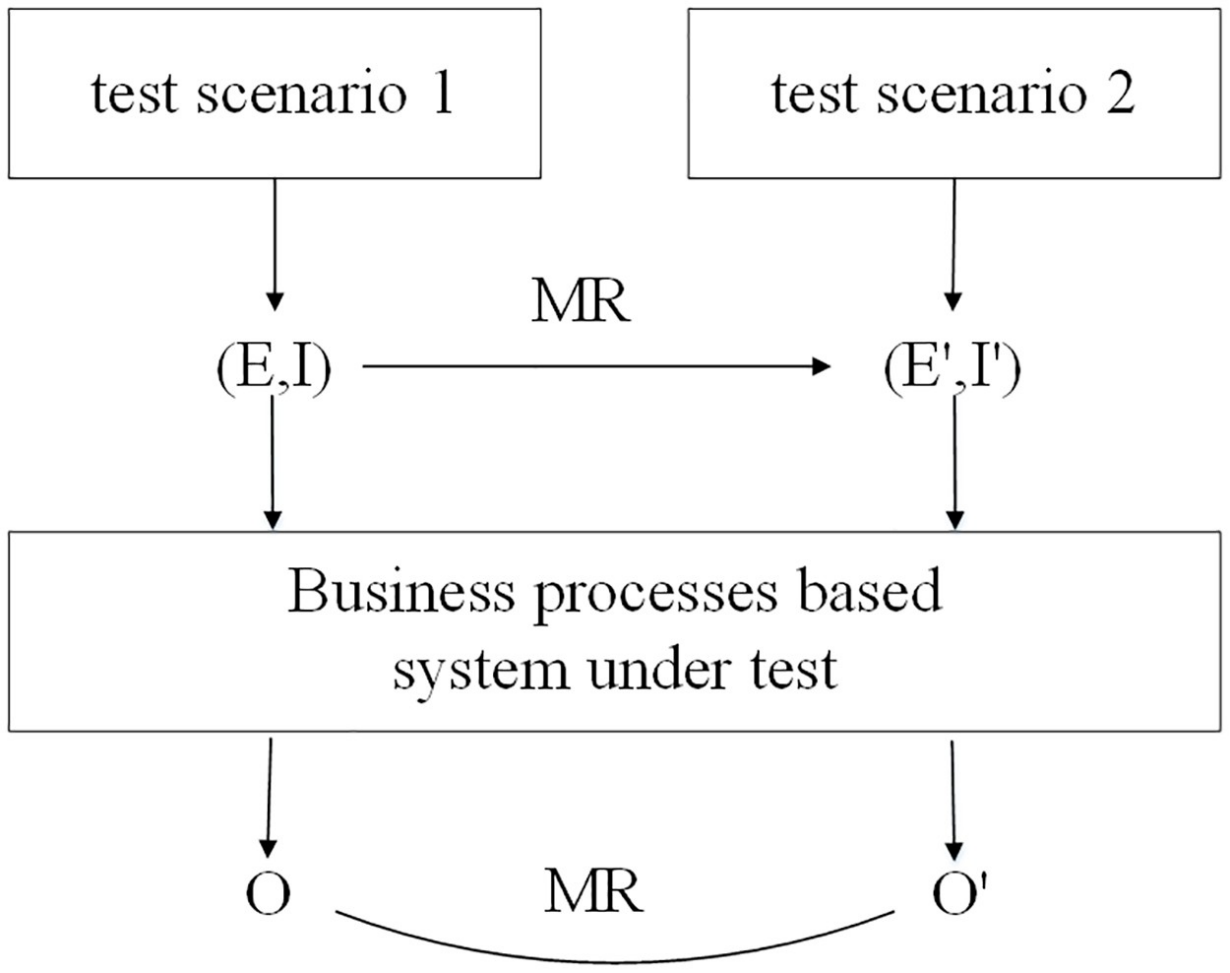
The procedure of testing business processes via MTES.

To test business-process-based software systems, we propose a method of metamorphic testing for event sequences (MTES) without using test oracles. In contrast to traditional MT, MTES focuses on the testing of business processes with not only input and output sequences but also event sequences. Therefore, the procedure of testing business processes by MTES in Fig 6 is slightly different from that of traditional MT. In general, the process includes the following steps.

- Test scenarios are identified from the business processes of a system, and event sequences are generated. Each event sequence represents a test scenario of a business process.
- An MR between event sequences is designed based on the properties of the event sequences, input sequences and output sequences.
- The source test case (*E*, *I*) is generated. *E* is one of these event sequences. *I* is the input sequence triggering the event sequence *E*, which can be generated by random testing [33] and fault-based testing [7].
- The follow-up test case (*E*′, *I*′) is constructed based on the source test case (*E*, *I*) and the given MR. *E*′ and *E* can be identical or not. *I*′ is the input sequence triggering the event sequence *E*′.
- Two test cases are executed in the system, and the corresponding output sequences, *O* and *O*′, are tested to check whether they violate the MR. If the MR is violated, the tested business processes are faulty. Note that testers can compare the ultimate outputs or the intermediate outputs and all outputs or partial outputs of the source and follow-up output sequences.

The key issue in MTES is to identify the metamorphic relation between event sequences. Compared with a traditional MR, a metamorphic relation between event sequences involves not only the properties among multiple input sequences and output sequences but also the properties between event sequences. We propose general rules to construct the follow-up event sequences, which are the properties of the event sequences listed in Table 1.

**Table 1:** Properties of event sequences.

Metamorphic properties	Involved event
Inserting one or multiple events	Correlative event
Deleting one or multiple events	Any appropriate event
Replacing one or multiple events	Any correlative and appropriate event
Permuting one or multiple events	Any appropriate event

A metamorphic relation between event sequences is defined as follows: if there exists one relation *R*_*I*_ between (*E*, *I*) and (*E*′, *I*′) and another relation *R*_*O*_ between *O* and *O*′, *R*_*O*_ is always satisfied whenever *R*_*I*_ is satisfied. This metamorphic relation can be presented in the following form.

$$\begin{array}{c} \text{MR}:R_{I}\left\{\left(E,I\right),\left(E^{{}^{\prime }},I^{{}^{\prime }}\right)\right\}\to R_{O}\left\{O,O^{{}^{\prime }}\right\} \end{array}$$

*E* = 〈*e*_1_, …, *e*_*n*_〉 is called the source event sequence, in which any two events *e*_*i*_ ∈ *E* and *e*_*j*_ ∈ *E* may refer to the same or different events. $E^{{}^{\prime }}=\langle e_{1}^{{}^{\prime }},...,e_{m}^{{}^{\prime }}\rangle$ is called the follow-up event sequence, where the events inside *E*′ and those inside *E* may be overlapped or not overlapped. *I* = 〈*I*_1_, …, *I*_*n*_〉 is called the source input sequence, where *I*_*i*_ can be derived from the input of the current event *e*_*i*_, the output of the previous event *e*_*i*−1_ or their combination. $I^{{}^{\prime }}=\langle I_{1}^{{}^{\prime }},...,I_{m}^{{}^{\prime }}\rangle$ is called the follow-up input sequence. *O* = 〈*O*_1_, …, *O*_*n*_〉 is called the source output sequence, and $O^{{}^{\prime }}=\langle O_{1}^{{}^{\prime }},...,O_{n}^{{}^{\prime }}\rangle$ is called the follow-up output sequence. Furthermore, the source and follow-up event sequences may be a single event, or an event sequence with multiple events or a combination of them. Therefore, MRs for event sequences can be categorized into the following three types according to the operations used to construct the follow-up test cases:

MR based on a fixed single-event sequence: $\left(E,I\right)\overset{E=E^{{}^{\prime }},\left|E\right|=1,I\neq I^{{}^{\prime }}}{\to }\left(E^{{}^{\prime }},I^{{}^{\prime }}\right)$
MR based on a fixed multi-event sequence: $\left(E,I\right)\overset{E=E^{{}^{\prime }},\left|E\right|>1,I\neq I^{{}^{\prime }}}{\to }\left(E^{{}^{\prime }},I^{{}^{\prime }}\right)$
MR based on varied event sequences: $\left(E,I\right)\overset{E\neq E^{{}^{\prime }},I\neq I^{{}^{\prime }}}{\to }\left(E^{{}^{\prime }},I^{{}^{\prime }}\right)$

Consider the example in Fig 5. We obtain an event sequence and construct the metamorphic relation MR3. In this MR, the source event sequence *E*_*s*_ is denoted as ‘calculate the weekly sales amount, calculate the weekly sales commission, calculate the total sales commission’, and the source input sequence *I*_*s*_ is a set *S* of all sales amounts from all salesmen. The follow-up event sequence can be constructed as (*E*_*f*_, *I*_*f*_), where the follow-up event sequence *E*_*f*_ is the same as the source event sequence *E*_*s*_, and the values of all elements in the follow-up input sequence *I*_*f*_ increase by the constant *m* than those in the source input sequence *I*_*s*_. Certainly, testers can also select single events or varied event sequences to test the system more comprehensively.

## Case studies

The experimental dataset used in this paper can be obtained from any of these URLs:

http://doi.org/10.6084/m9.figshare.5349901
https://figshare.com/s/164baa1941739b712971
https://figshare.com/articles/Dataset_for_the_article_A_Metamorphic_Testing_Approach_for_Event_Sequences_/5349901.

### Experimental setup

According to the MTES procedure, we conduct three case studies to illustrate our approach and validate its effectiveness. The software systems include a simplified electricity bill payment system in case study 1, a simplified interbank transaction system in case study 2 and an elastic cloud management system in case study 3. Cases 1 and 2 test various business processes. Some of the processes involve multistep calculations based on different algorithms and data transfer for which it is expensive and error-prone to obtain test oracles, as shown in example 2 in the running examples. Although other processes can implement test oracles, they must be tested with a substantial quantity of test data due to critical transactions. MTES is easy and low-cost for this type of critical transaction without the need to calculate test outputs. Case 3 tests a complex autoscaling process of a virtual cluster, which is related to not only the elastic cloud management system but also the Openstack cloud platform. A test oracle is not available for this process because of the unpredictable resource utilization of this cluster. Therefore, we use MTES to test the autoscaling mechanism of a virtual cluster. In the experimental procedure, the following common methods are used to setup the experiments.

#### Test case generation

In terms of event sequence generation, we use an ESG to manually generate the source event sequences based on basis path testing. The follow-up event sequences are constructed based on the source event sequences and some of the related properties. In our case studies, we select key event sequences to implement the experiments. These event sequences are sufficient to illustrate our approach. For each MR, we use the random testing technique to generate the source input sequence based on the source event sequence. Thus, we can combine the source input sequence with the source event sequence to generate the source test case. Then, the follow-up test case can be constructed based on the MR and the source test case. Thus, a series of test groups (each of which includes a source test case and a follow-up test case) are composed. In the following case studies, some constraints exist in the test groups.

- The numerical inputs involving money, such as the transaction amount and the balance, must be positive. The numerical outputs involving money can be made negative by setting the parameters of these systems to compare the mathematical relations between the source and follow-up outputs.
- All the inputs and outputs involving money must keep two digits after the decimal point. This means that rounding is used in the calculations involving money.
- The transaction amount of an ATM withdrawal in this paper cannot exceed 5000 and must be a multiple of 50. The transaction amount of any deposit cannot exceed 200000.
- With respect to an event sequence, the input of an event derived from the output of the previous event is also affected by the input of the previous event.

#### Mutant generation

The mutation analysis technique applies mutation operators to inject faults into a program and thus generates various mutants to evaluate the effectiveness of a test method. A mutant is generally a program with one statement or expression mutated by a mutation operator. If a mutant exhibits a behavior different from the SUT, the mutant is killed, and the fault is detected. Mutants generated by mutation operators are similar to real faults [38]. We use the mujava [39] tool to automatically generate mutants for the program under test. Mujava provides two types of mutation operators: method-level operators and class-level operators. In this paper, we focus on faults for which incorrect outputs are produced, such as errors in calculation, logic and conditions. Therefore, we use only a few method-level operators (arithmetic, relational and conditional operators) to generate mutants. Each mutant is a program with one mutated statement. An equivalent mutant is a mutated program that is behaviorally equivalent to the original and cannot be killed by any test case. We select only killable mutants (i.e., non-equivalent mutants [39, 40]), excluding the mutants that cause crashes, exceptions and obvious errors in case studies 1 and 2. Because the system in case study 3 is implemented in the Javascript and Python languages, mutants cannot be generated automatically by mutation tools. Three different program versions with real faults are provided to evaluate the effectiveness of our approach in case study 3.

#### Effective measurement

Clearly, the MTES we propose is feasible in theory, but its effectiveness requires further validation in practical applications. We conduct three case studies to investigate this issue in terms of two metrics.

The first metric is the mutation score (MS), which is an intuitive indicator of the effectiveness of MT and is defined as follows:

$$\begin{array}{c} MS=\frac{N_{k}}{N_{n}} \end{array}$$

where *N*_*k*_ denotes the number of killed mutants and *N*_*n*_ denotes the number of all non-equivalent mutants. The second metric is the fault-detection rate, which is defined as follows:

$$\begin{array}{c} FDR=\frac{N_{v}}{N_{a}} \end{array}$$

where *N*_*v*_ denotes the number of test cases that cause their outputs to violate an MR and *N*_*a*_ denotes the total number of test cases.

We adopt MS as the metric to assess the effectiveness of our approach in case studies 1 and 2. Because mutation analysis is not used in case study 3, we use *FDR* as the metric in case study 3. This metric can more realistically reflect the effectiveness of our approach because of the real faults in this case. To compare the source and follow-up output results, we write scripts to automatically determine whether they violate the MRs.

#### Imprecision

The problem of imprecision arises when test outputs are compared. A loss of precision occurs in floating-point operations for Java, which can cause test outputs to violate an MR even if the test outputs are actually correct. In addition, rounding errors can also cause false positives. For example, the transaction fee of a deposit is calculated based on the formula 0.001 * *A*, where *A* denotes the deposit amount. If we deposit 4124.23 onto a card with a balance of 2000.00 in an MR, we will achieve a new balance 6120.11. If we deposit 8248.46 onto a card with a balance 4000.00, the new balance should theoretically change to double the previous output. However, the actual result is only 12240.21 due to a rounding error. We may incorrectly think the program is faulty because the outputs violate the MR. These problems are solved by setting thresholds in the comparison of test outputs such that no violation is reported if the difference in test outputs is within the threshold.

### Case study 1

#### A simplified electricity bill payment system

**Fig 7:**
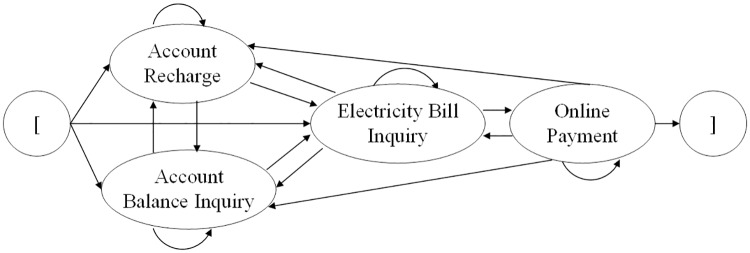
ESG of a simplified electricity bill payment system.

Fig 7 shows the ESG of a simplified electricity bill payment system from a community. Four main events (i.e., functions) are included: account balance inquiry, account recharge, electricity bill inquiry and online payment. The implementation of these functions consists of 180 lines of core code written in Java that mainly achieve numerical calculations of these functions, connection to a MySQL database and SQL queries. When a consumer logs into this system, he can check his account balance by implementing the event ‘account balance inquiry’. To increase his account balance, he can also deposit money into his account by implementing the event ‘account recharge’. Furthermore, he can obtain his electricity bill to know his monthly electricity fee by implementing the event ‘electricity bill inquiry’. The monthly electricity fee is calculated using the electricity price and the monthly electricity consumption of a consumer. Then, the fee is deducted from his account balance by implementing the event ‘online payment’. The event ‘online payment’ cannot be executed until the event ‘electricity bill inquiry’ is implemented successfully. The classes of electricity prices are shown in Table 2. The electricity price *E*_*p*_ varies with the number of family members *F*_*m*_ and the cumulative annual electricity consumption *C*_*ca*_, which is the total amount of electricity consumed by a consumer in one year. According to this price table, each family pays the electricity bill from their online account monthly. In December of each year, a low-income family is compensated by CNY98.45, that is, CNY98.45 is deposited into its account.

**Table 2:** Electricity price classes.

*F*_*m*_	*C*_*ca*_(*kWh*)	*E*_*p*_(*CNY*)
*F*_*m*_ < 5	*C*_*ca*_ ≤ 2520	*E*_*p*_ = 0.5469
*F*_*m*_ < 5	2520 < *C*_*ca*_ ≤ 4800	*E*_*p*_ = 0.5969
*F*_*m*_ ≥ 5	*C*_*ca*_ ≤ 3720	*E*_*p*_ = 0.5469
*F*_*m*_ ≥ 5	3720 < *C*_*ca*_ ≤ 4800	*E*_*p*_ = 0.5969
*F*_*m*_ < 5 or *F*_*m*_ ≥ 5	*C*_*ca*_ > 4800	*E*_*p*_ = 0.8469

The input of an account recharge is the 2-tuple (*N*, *A*), where *N* denotes the account number and *A* denotes the recharge amount. The input of an account balance inquiry is a user’s account number *N*. The outputs of an account recharge and an account balance inquiry are both denoted as (*N*, *B*), where *B* is the new balance. The input of an electricity bill inquiry is the 2-tuple (*N*, *M*), and its output is the 5-tuple (*N*, *M*, *C*_*m*_, *F*, *C*_*a*_), where *M* denotes the month considered, *C*_*m*_ denotes the monthly electricity consumption, *C*_*a*_ denotes the annual electricity consumption, the electricity fee *F* is calculated using the formula *F* = *E*_*p*_ * *C*_*m*_, and the cumulative annual electricity consumption *C*_*ca*_ in Table 2 is obtained based on the formula *C*_*ca*_ = *C*_*m*_ + *C*_*a*_. The input of an online payment is the output of an electricity bill inquiry. The new balance *B* from the output (*N*, *B*) of an online payment is calculated using the formula *B* = *B*_*o*_ − *C*_*m*_, where *B*_*o*_ is the balance before paying the electricity bill.

#### Metamorphic relations of a simplified electricity bill payment system

We create accounts *N*_1_, *N*_2_, *N*_3_, and *N*_4_ with the same balance *B*_0_ for normal-income families with three members, four members, five members and six members, respectively. Account *N*_5_ with balance *B*_0_ + *M* is for a normal-income family with five members. Account *N*_6_ with balance *B*_0_ is for a low-income family with three members. To design MRs between event sequences, the following basic properties of this system are first identified.

- For each family, the electricity fee *F* and the new balance *B* after online payment are calculated monthly based on the formulas *F* = *E*_*p*_ * *C*_*m*_ and *B* = *B*_*o*_ − *F*.
- A low-income family will be compensated CNY98.45 in December of each year. That is, the new balance of a low-income family is calculated using the formula *B* = *B*_*o*_ − *F* + 98.45 in December of each year.

Some event sequences are identified from the ESG of the electricity bill payment system shown in Fig 7, such as ‘Account Recharge’, ‘Electricity Bill Inquiry’, and 〈Account Recharge,Electricity Bill Inquiry,Online Payment〉. Based on these event sequences and basic properties, we construct different types of MRs.

1. MR based on a fixed single-event sequence. For a fixed single-event sequence *e*, the source and follow-up test cases can be described as (*E*_*s*_, *I*_*s*_) and (*E*_*f*_, *I*_*f*_), where the source event sequence is the same as the follow-up event sequence, that is, *E*_*s*_ = *E*_*f*_ = *e*. Correspondingly, their output sequences are expressed as *O*_*s*_ and *O*_*f*_. In this paper, the characters ‘s’ and ‘f’ in the subscript denote ‘source’ and ‘follow-up’, respectively.
  **MR1:** For the fixed single-event sequence ‘Account Recharge’, if the source input sequence is denoted as *I*_*s*_ = Account Recharge(*N*_1_, *A*), then we can construct a follow-up input sequence *I*_*f*_ = Account Recharge(*N*_2_, *A* + *C*) by adding a positive integer *C* to the recharge amount *A* and changing the account number from *N*_1_ to *N*_2_. Denote the source and follow-up outputs as *O*_*s*_ = (*N*_1_, *B*_*s*_) and *O*_*f*_ = (*N*_2_, *B*_*f*_), where *B*_*s*_ and *B*_*f*_ represent the new balances of the source and follow-up outputs; thus, we will obtain the output relation *B*_*f*_ = *B*_*s*_ + *C*.
  **MR2:** For a fixed single-event sequence ‘Electricity Bill Inquiry’, if the source input sequence is represented as *I*_*s*_ = Electricity Bill Inquiry(*N*_1_, 5), where the input parameter ‘5’ means May, then we can construct a follow-up input sequence *I*_*f*_ = Electricity Bill Inquiry(*N*_2_, 5) by changing the account number *N*_1_ to *N*_2_. Denote the source and follow-up output sequences as *O*_*s*_ = (*N*_1_, 5, *C*_*ms*_, *F*_*s*_, *C*_*as*_) and *O*_*f*_ = (*N*_2_, 5, *C*_*mf*_, *F*_*f*_, *C*_*af*_). If the follow-up monthly electricity consumption *C*_*mf*_ is twice as large as the source electricity consumption *C*_*ms*_ and both the source and follow-up cumulative annual electricity consumptions are not more than 2520 kWh, that is, *C*_*as*_ + *C*_*ms*_ < = 2520 and *C*_*af*_ + *C*_*mf*_ < = 2520, the follow-up electricity fee *F*_*f*_ should be twice as large as the source electricity fee *F*_*s*_.
2. MR based on a fixed multi-event sequence. Suppose the source and follow-up test cases are (*E*_*s*_, *I*_*s*_) and (*E*_*f*_, *I*_*f*_), where the source event sequence *E*_*s*_ is the same as the follow-up event sequence *E*_*f*_ with multiple events, that is, *E*_*s*_ = *E*_*f*_ = 〈*e*_1_, …, *e*_*n*_〉. *I*_*s*_ and *I*_*f*_ are the source and follow-up input sequences of this event sequence, and their output sequences are represented as *O*_*s*_ and *O*_*f*_.
  Given the fixed multi-event sequence 〈Account Recharge, Electricity Bill Inquiry, Online Payment〉 and the source input sequence *I*_*s*_ = 〈Account Recharge(*N*_1_, *A*), Electricity Bill Inquiry(*N*_1_, 5), Online Payment(*N*_1_, 5, *C*_*ms*_, *F*_*s*_, *C*_*as*_)〉, the follow-up input sequence *I*_*f*_ = 〈Account Recharge(*N*_2_, *A* + *K*), Electricity Bill Inquiry(*N*_2_, 5), Online Payment(*N*_2_, 5, *C*_*ms*_ + *C*, *F*_*f*_, *C*_*af*_)〉 can be constructed by changing the account number from *N*_1_ to *N*_2_, separately adding the positive integers *K* and *C* to the recharge amount *A* and the monthly electricity consumption *C*_*ms*_, and changing the monthly electricity fee from *F*_*s*_ to *F*_*f*_ and the annual electricity consumption from *C*_*as*_ to *C*_*af*_. Thus, the corresponding source and follow-up output sequences can be denoted as $O_{s}=\langle \left(N_{1},B_{s}^{1}\right),\left(N_{1},5,C_{ms},F_{s},C_{as}\right),\left(N_{1},B_{s}\right)\rangle$ and $O_{f}=\langle \left(N_{2},B_{f}^{1}\right),(N_{2},5,C_{ms}+C,F_{f},C_{af}),\left(N_{2},B_{f}\right)\rangle$, where $B_{s}^{1}$ and $B_{f}^{1}$ are the source and follow-up balances after executing the first event ‘Account Recharge’ and *B*_*s*_ and *B*_*f*_ are the final source and follow-up balances for card number *N*_1_ and card number *N*_2_. Thus, we can design the metamorphic relations *MR*3-*MR*4.
  **MR3:** If both the source and follow-up cumulative annual electricity consumption are within the range (0, 2520], that is, *C*_*as*_ + *C*_*ms*_ < = 2520 and *C*_*af*_ + *C*_*mf*_ < = 2520, then the follow-up final balance *B*_*f*_ should satisfy the relation *B*_*f*_ = *B*_*s*_ + *K* − 0.5469*C*.
  **MR4:** If the source cumulative annual electricity consumption *C*_*ms*_ + *C*_*as*_ is within the range (0, 2520] and the follow-up annual electricity consumption *C*_*af*_ is within the range (4800, + ∞), we will obtain the following output relation for the follow-up final balance: *B*_*f*_ = *B*_*s*_ + *K* − 0.3 * *C*_*ms*_ − 0.8469*C*.
  **MR5:** Supposing that the source input sequence is denoted as *I*_*s*_ = 〈Account Recharge(*N*_3_, *A*), Electricity Bill Inquiry(*N*_3_, 5), Online Payment(*N*_3_, 5, *C*_*ms*_, *F*_*s*_, *C*_*as*_)〉, the follow-up input sequence *I*_*f*_ = 〈Account Recharge(*N*_4_, *A*), Electricity Bill Inquiry(*N*_4_, 5), Online Payment(*N*_4_, 5, *C*_*ms*_ + *C*, *F*_*f*_, *C*_*af*_)〉 can be constructed by changing account number *N*_3_ with five family members to account number *N*_4_ with six family members, adding a positive integer *C* to the monthly electricity consumption *C*_*ms*_, changing the monthly electricity fee from *F*_*s*_ to *F*_*f*_ and changing the annual electricity consumption from *C*_*as*_ to *C*_*af*_. Supposing the source annual electricity consumption *C*_*as*_ > 2520, the source cumulative annual electricity consumption *C*_*as*_ + *C*_*ms*_ < = 3720, the follow-up annual electricity consumption *C*_*af*_ > 3720 and the follow-up cumulative annual electricity consumption *C*_*af*_ + *C*_*ms*_ + *C* < = 4800, the output sequences should satisfy the relation *B*_*f*_ = *B*_*s*_ − 0.05 * *C*_*ms*_ − 0.5969 * *C*.
  **MR6:** Supposing that the source input sequence is described as *I*_*s*_ = 〈Account Recharge(*N*_2_, *A*), Electricity Bill Inquiry(*N*_2_, 5), Online Payment(*N*_2_, 5, *C*_*ms*_, *F*_*s*_, *C*_*as*_)〉, the follow-up input sequence *I*_*f*_ = 〈Account Recharge(*N*_3_, *A* + *K*), Electricity Bill Inquiry(*N*_3_, 5), Online Payment(*N*_3_, 5, 2 * *C*_*ms*_, *F*_*f*_, *C*_*af*_)〉 can be constructed by changing account number *N*_2_ with four members to account number *N*_3_ with five members, adding a positive integer *K* to the recharge amount *A*, multiplying the monthly electricity consumption *C*_*ms*_ by a positive integer 2, changing the electricity fee from *F*_*s*_ to *F*_*f*_ and changing the annual electricity consumption from *C*_*as*_ to *C*_*af*_. If there exist a source annual electricity consumption *C*_*as*_ > 2520, a source cumulative annual electricity consumption *C*_*as*_ + *C*_*ms*_ < = 4800 and a follow-up annual electricity consumption *C*_*af*_ > 4800, we can obtain the following output relation for the follow-up final balance: *B*_*f*_ = *B*_*s*_ + *K* − 1.0969 * *C*_*ms*_.
  Given the fixed multi-event sequence *E*_*s*_ = *E*_*f*_ = 〈Electricity Bill Inquiry, Online Payment〉, the source and follow-up output sequences are denoted as *O*_*s*_ = 〈(*N*_*s*_, *M*_*o*_, *C*_*ms*_, *F*_*s*_, *C*_*as*_), (*N*_*s*_, *B*_*s*_)〉 and *O*_*f*_ = 〈(*N*_*f*_, *M*_*o*_, *C*_*mf*_, *F*_*f*_, *C*_*af*_), (*N*_*f*_, *B*_*f*_)〉, where *M*_*o*_ denotes the month considered. Supposing an account *N*_7_ with balance *B*_0_ + *M* is from a normal-income family with three members, we can construct the following two MRs.
  **MR7:** Given the source input sequence *I*_*s*_ = 〈Electricity Bill Inquiry(*N*_1_, 5), Online Payment(*N*_1_, 5, *C*_*ms*_, *F*_*s*_, *C*_*as*_)〉, the follow-up input sequence *I*_*f*_ = 〈Electricity Bill Inquiry(*N*_7_, 5), Online Payment(*N*_7_, 5, *C*_*ms*_ + *C*, *F*_*f*_, *C*_*af*_)〉 can be constructed by changing account number *N*_1_ with balance *B*_0_ to account number *N*_7_ with balance *B*_0_ + *M*, adding a positive integer *C* to the monthly electricity consumption *C*_*ms*_, changing the electricity fee from *F*_*s*_ to *F*_*f*_ and changing the annual electricity consumption from *C*_*as*_ to *C*_*af*_. If the source and follow-up cumulative annual electricity consumptions are both within the range (0, 2520], that is, *C*_*as*_ + *C*_*ms*_ < = 2520 and *C*_*af*_ + *C*_*ms*_ + *C* < = 2520, we can obtain the following output relation: *B*_*f*_ = *B*_*s*_ + *M* − 0.5469*C*.
  **MR8:** Given the source input sequence *I*_*s*_ = 〈Electricity Bill Inquiry(*N*_6_, 12), Online Payment(*N*_6_, 12, *C*_*ms*_, *F*_*s*_, *C*_*as*_)〉, the follow-up input sequence *I*_*f*_ = 〈Electricity Bill Inquiry(*N*_7_, 12), Online Payment(*N*_7_, 12, *C*_*ms*_ + *C*, *F*_*f*_, *C*_*af*_)〉 can be constructed by changing account number *N*_6_ with low income and balance *B*_0_ to account number *N*_7_ with normal income and balance *B*_0_ + *M*, adding a positive integer *C* to the monthly electricity consumption *C*_*ms*_, changing the electricity fee from *F*_*s*_ to *F*_*f*_ and changing the annual electricity consumption from *C*_*as*_ to *C*_*af*_. If the source and follow-up cumulative annual electricity consumptions are both within the range (0, 2520], that is, *C*_*as*_ + *C*_*ms*_ < = 2520 and *C*_*af*_ + *C*_*ms*_ + *C* < = 2520, we will achieve the following output relation for the follow-up final balance: *B*_*f*_ = *B*_*s*_ + *M* − 0.5469*C* − 98.45.
3. MR based on varied event sequences. For varied event sequences, the source and follow-up test cases are denoted as (*E*_*s*_, *I*_*s*_) and (*E*_*f*_, *I*_*f*_), where *E*_*s*_ ≠ *E*_*f*_, and the corresponding output sequences are denoted as *O*_*s*_ and *O*_*f*_. Assume that an account *N*_8_ with balance *B*_0_ + 2*M* is from a normal-income family with three members, we can construct MRs based on varied event sequences as follows.
  **MR9:** The account recharge event is a loop event. If we recharge the amount *A* + *B* into an account once, the same balance can be obtained as would be obtained if it were recharged by the amounts *A* and *B* sequentially. Supposing that the source event sequence and input sequence are separately denoted as *E*_*s*_ = Account Recharge and *I*_*s*_ = Account Recharge(*N*_1_, *A* + *B*), we can construct the follow-up event sequence *E*_*f*_ = 〈Account Recharge, Account Recharge〉 and the follow-up input sequence *I*_*f*_ = 〈Account Recharge(*N*_1_, *A*), Account Recharge(*N*_1_, *B*)〉. Then, their output sequences should have the same balance.
  For *E*_*s*_ = 〈Account Recharge, Electricity Bill Inquiry, Online Payment〉, we can construct the metamorphic relations *MR*10-*MR*13.
  **MR10:** Suppose that the follow-up event sequence is constructed by replacing the event ‘Account Recharge’ of the source event sequence with the event ‘Account Balance Inquiry’. Given the source input sequence *I*_*s*_ = 〈Account Recharge(*N*_1_, *M*), Electricity Bill Inquiry(*N*_1_, 5), Online Payment(*N*_1_, 5, *C*_*ms*_, *F*_*s*_, *C*_*as*_)〉, where *M* is the recharge amount, the follow-up input sequence *I*_*f*_ = 〈Account Balance Inquiry(*N*_8_), Electricity Bill Inquiry(*N*_8_, 5), Online Payment (*N*_8_, 5, *C*_*ms*_ + *C*, *F*_*f*_, *C*_*af*_)〉 is constructed by changing account number *N*_1_ with balance *B*_0_ to account number *N*_8_ with balance *B*_0_ + 2*M*, adding a positive integer *C* to the monthly electricity consumption *C*_*ms*_, changing the electricity fee from *F*_*s*_ to *F*_*f*_ and changing the annual electricity consumption from *C*_*as*_ to *C*_*af*_. The source and follow-up output sequences are expressed as $O_{s}=\langle \left(N_{1},B_{s}^{1}\right),\left(N_{1},5,C_{ms},F_{s},C_{as}\right),\left(N_{1},B_{s}\right)\rangle$ and $O_{f}=\langle \left(N_{8},B_{f}^{1}\right),\left(N_{8},5,C_{ms}+C,F_{f},C_{af}\right),\left(N_{8},B_{f}\right)\rangle$, where $B_{s}^{1}$ and $B_{f}^{1}$ refer to the first source and follow-up balances, and *B*_*s*_ and *B*_*f*_ refer to the final source and follow-up balances in the source and follow-up output sequences. If the source and follow-up cumulative annual electricity consumptions are both within the range (0, 2520], that is, *C*_*as*_ + *C*_*ms*_ < = 2520 and *C*_*af*_ + *C*_*ms*_ + *C* < = 2520, we can obtain the relation with the follow-up final balance *B*_*f*_ = *B*_*s*_ + *M* − 0.5469 * *C*.
  **MR11:** Compared with *MR*10, *MR*11 uses account number *N*_3_ with balance *B*_0_ and account number *N*_5_ with balance *B*_0_ + *M*, and has different relations between the source and follow-up input sequences, that is, *C*_*ms*_ > 2520, *C*_*as*_ + *C*_*ms*_ < = 3720, *C*_*af*_ > 3720 and *C*_*af*_ + *C*_*ms*_ + *C* < = 4800. Thus, the source and follow-up output sequences should satisfy the relation with the follow-up final balance *B*_*f*_ = *B*_*s*_ − 0.05 * *C*_*ms*_ − 0.5969 * *C*.
  **MR12:** Based on the source event sequence *E*_*s*_, the follow-up event sequence *E*_*f*_ = Account Recharge is constructed by deleting the events ‘Electricity Bill Inquiry’ and ‘Online Payment’ from the source event sequence. If the source input sequence is described as *I*_*s*_ = 〈Account Recharge(*N*_1_, *A*), Electricity Bill Inquiry(*N*_1_, 5), Online Payment(*N*_1_, 5, *C*_*ms*_, *F*_*s*_, *C*_*as*_)〉, the follow-up input sequence *I*_*f*_ = Account Recharge(*N*_2_, *A* + *K*) can be constructed by changing the account number from *N*_1_ to *N*_2_, adding a positive integer *K* to the recharge amount *A*, changing the electricity fee from *F*_*s*_ to *F*_*f*_ and changing the annual electricity consumption from *C*_*as*_ to *C*_*af*_. The corresponding source and follow-up output sequences are separately denoted as $O_{s}=\langle \left(N_{1},B_{s}^{1}\right),\left(N_{1},5,C_{ms},F_{s},C_{as}\right),\left(N_{1},B_{s}\right)\rangle$ and *O*_*f*_ = (*N*_2_, *B*_*f*_). If the source cumulative annual electricity consumption is within the range (0, 2520], that is, *C*_*as*_ + *C*_*ms*_ < = 2520, the output relation *B*_*f*_ = *B*_*s*_ + *K* + 0.5469 * *C*_*ms*_ should be setup.
  **MR13:** Based on the source event sequence *E*_*s*_, the follow-up event sequence *E*_*f*_ = 〈Electricity Bill Inquiry, Online Payment, Account Recharge〉 is constructed by permuting the order of events in the source event sequence. Given the source input sequence *I*_*s*_ = 〈Account Recharge(*N*_1_, *A*), Electricity Bill Inquiry(*N*_1_, 5), Online Payment(*N*_1_, 5, *C*_*ms*_, *F*_*s*_, *C*_*as*_)〉, the follow-up input sequence *I*_*f*_ = 〈Electricity Bill Inquiry(*N*_2_, 5), Online Payment(*N*_2_, 5, *C*_*ms*_, *F*_*s*_, *C*_*as*_), Account Recharge(*N*_2_, *A*)〉 is constructed by changing account number *N*_1_ with three members to account number *N*_2_ with four members. If the cumulative annual electricity consumption is within the range (0, 2520], that is, *C*_*as*_ + *C*_*ms*_ < = 2520, the source and follow-up final balances should be the same.

#### Experimental results and analysis

We use mutation analysis to generate 548 mutants excluding the equivalent mutants and those that lead to exceptions, crashes and obvious errors. Furthermore, we generate 200 test groups (each group includes one source and one follow-up test case) for each MR. All test groups are executed, and their output sequences are compared. MSs are calculated, and the results are shown in Table 3. We obtain the following findings.

**Table 3:** Mutation scores of MRs for all mutants.

MR1	MR2	MR3	MR4	MR5	MR6	MR7
0.91%	6.02%	7.30%	9.49%	16.24%	13.87%	6.39%
MR8	MR9	MR10	MR11	MR12	MR13	All MRs
12.96%	1.28%	7.85%	16.79%	10.22%	3.28%	39.23%

1. Combined, the MRs kill 39.23% of all mutants. Each MR kills a different number of mutants, which indicates different fault-detection capability. *MR*11 is the strongest and kills 16.79% of all mutants, whereas the weakest metamorphic relation, *MR*1, kills only 0.91%. *MR*2 is more effective than *MR*1 for fixed single-event sequences, *MR*5 has a higher MS than other MRs for fixed multi-event sequences, and *MR*11 has stronger fault-detection capability than other MRs for varied event sequences. Intuitively, MRs with more events are more effective than those that have only a single event. For example, *MR*3-*MR*12 kill more mutants than do *MR*1 and *MR*2. Although some MRs are constructed based on the same fixed multi-event sequence, they also show different fault-detection capabilities. For instance, *MR*3-*MR*6 have different MSs. The most effective metamorphic relation, *MR*5, kills 16.24% of all mutants, whereas the least effective one, *MR*3, kills only 7.30%.
2. An MR based on varied event sequences normally has higher fault-detection capability than an MR based on a fixed event sequence. Certainly, there is a precondition that they have the same source event sequence and input sequence but different follow-up event sequences and input sequences. For example, *MR*10 and *MR*12 are more effective than *MR*3 due to the different follow-up event sequences and input relations. Likewise, *MR*11 is more effective than *MR*5. *MR*9 is more effective than *MR*1 because *MR*9 continuously executes the event of account recharge twice rather than once, as in *MR*1. Furthermore, the MS of *MR*10 exceeds the sum of the MSs of *MR*1 and *MR*7 although the account balance inquiry event yields no mutants. This result occurs because *MR*10 has different event sequences for the source and follow-up test cases.
3. MRs with different input and output relations have different effectiveness. The effectiveness of MT for event sequences is also affected by factors other than the event sequences, such as the input and output relations. For instance, *MR*3-*MR*6 have different fault-detection capabilities due to different input and output relations even though they are derived from the same event sequence. *MR*7 and *MR*8 also exhibit different MSs due to different input and output relations. MRs with richer (i.e., more complex and different) input and output relations are more effective. For instance, *MR*5 and *MR*6 kill more mutants than do *MR*3 and *MR*4. *MR*11 has a higher MS than does *MR*10. *MR*13 has the lowest MS among *MR*10-*MR*13 due to having the weakest input and output relations.

To investigate the effectiveness of MRs in detail, we further analyze the results for different types of mutants. All mutants fall into three categories:

- mathematics mutants, in which the statements involving mathematical calculations are mutated by arithmetic operators, such as ‘+’ instead of ‘-’.
- off-by-one mutants, in which variables are adjusted by one, such as inserting ‘++’ before or after variables.
- condition mutants, in which the condition statements are mutated by relational operators or conditional operators, such as using ‘<’ instead of ‘>’ or inserting ‘!’ before a conditional expression.

We classify the mutants into 188 mathematics mutants, 167 off-by-one mutants and 193 condition mutants. The MSs of the MRs are presented with respect to mutant type in Table 4. Each MR has a different sensitivity to each type of mutants. *MR*1, *MR*3 and *MR*7 are not sensitive to off-by-one mutants, with an MS of 0%. Although *MR*3-*MR*6 are designed on the basis of the same event sequence, they have different sensitivities to different types of mutants. *MR*3 cannot kill any off-by-one mutant, and *MR*4 is sensitive to mathematics and condition mutants. *MR*5 and *MR*6 both have relatively high sensitivities to all types of mutants, with the MSs greater than 10%. *MR*8 presents higher sensitivity to all types of mutants than does *MR*7 due to its richer input relations. *MR*10 and *MR*12 kill more mathematics and off-by-one mutants than does *MR*3. *MR*9 kills the same number of mathematics mutants and more off-by-one mutants than does *MR*1. *MR*1 has the same source and follow-up event sequences, whereas *MR*9 goes through different event sequences. *MR*1, *MR*9 and *MR*13 cannot kill any condition mutant. No test cases from *MR*1 and *MR*9 go through the mutated statements in these condition mutants. *MR*13 executes some of the mutated statements but produces an MS of 0.00%. Therefore, we set ‘0.00%(*unreachable*)’ for *MR*1 and *MR*9. Overall, *MR*11 kills the most mutants and appears to have the strongest fault-detection capability for each type of mutant. Combined, the MRs kill 28.19% of the mathematics mutants, 31.74% of the off-by-one mutants and 56.48% of the condition mutants.

**Table 4:** Mutation scores of MRs for different types of mutants.

	Mathematics	Off-by-one	Condition
MR1	2.66%	0.00%	0.00%(unreachable)
MR2	2.13%	10.78%	5.70%
MR3	8.51%	0.00%	12.44%
MR4	10.64%	2.99%	13.99%
MR5	11.70%	14.37%	22.28%
MR6	12.23%	10.18%	18.65%
MR7	5.85%	0.00%	12.44%
MR8	11.17%	9.58%	17.62%
MR9	2.66%	1.20%	0.00%(unreachable)
MR10	9.04%	1.20%	12.44%
MR11	12.77%	14.97%	22.28%
MR12	10.64%	14.37%	6.22%
MR13	4.26%	5.99%	0.00%
All MRs	28.19%	31.74%	56.48%

**Fig 8:**
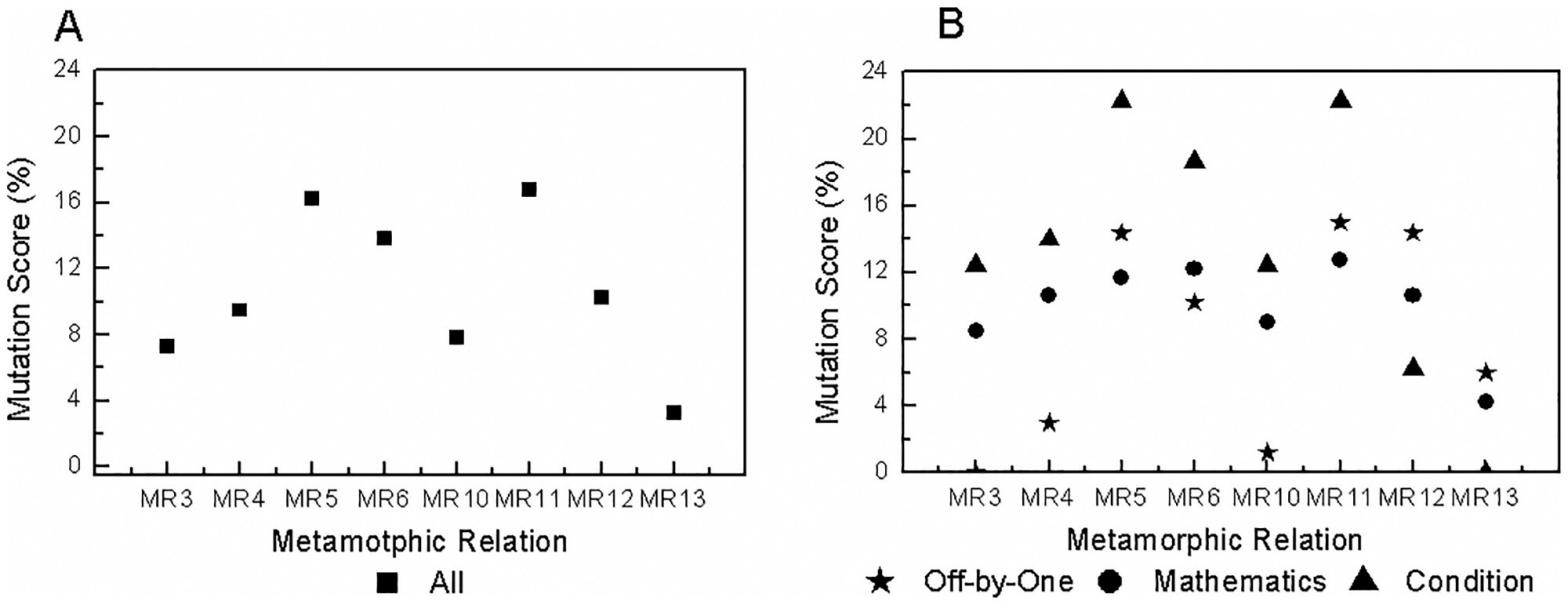
Mutation scores of the MRs based on the same source event sequence 〈Account Recharge, Electricity Bill Inquiry, Online Payment〉. A: For all mutants. B: For different types of mutants.

For clarity, we further investigate the MRs based on the same source event sequence 〈Account Recharge, Electricity Bill Inquiry, Online Payment〉. The results are shown in Fig 8. An MR designed by permutation, such as *MR*13, is insensitive in killing all types of mutants. MRs with addition in the input relations, such as *MR*3, *MR*4 and *MR*10, are weak in killing off-by-one mutants, with the MSs less than 3%. MRs with a greater number of different determination conditions (that is, execution paths), such as *MR*5, *MR*6 and *MR*11, are more sensitive to condition mutants. In general, MRs that are as different as possible have higher fault-detection capabilities and sensitivities for all types of mutants.

### Case study 2

#### A simplified interbank transaction system

**Fig 9:**
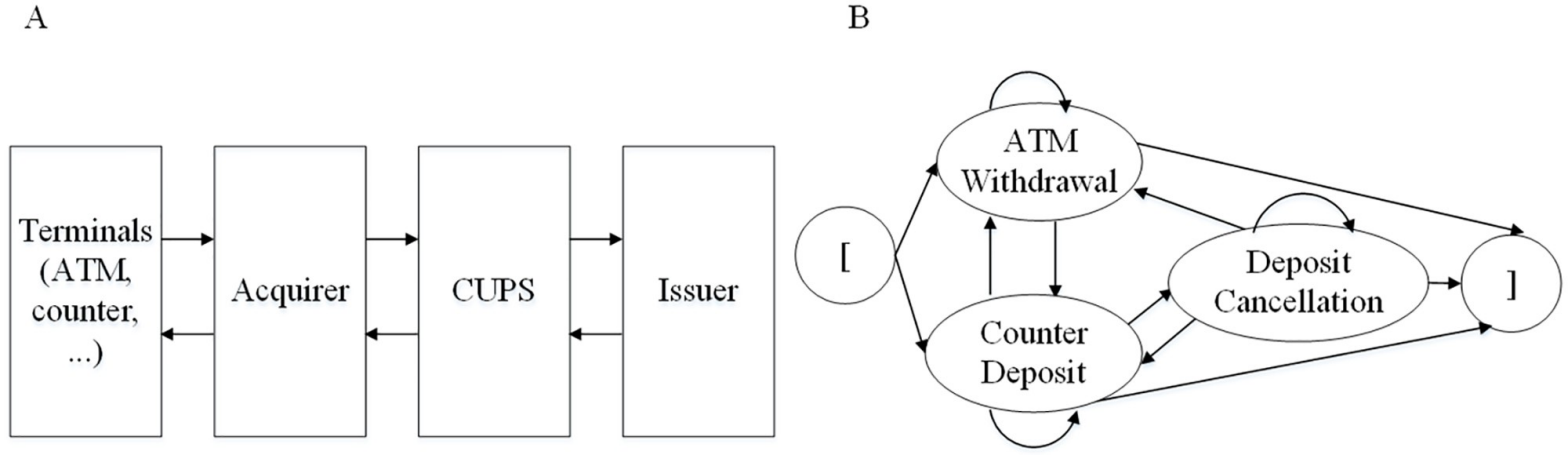
Process and ESG of interbank transactions. A:Process. B:ESG.

The process of interbank transactions is shown in Fig 9A. The acquirer (receiving bank) receives the card transaction details from various terminals and transmits them to the issuer through an intermediate process system (CUPS). The issuer (issuing bank) processes these transactions and replies to the acquirer. The system under test is a simplified program from the transaction process system of the issuer. Three main features are offered in Fig 9B: interbank ATM withdrawal, interbank counter deposit and deposit cancellation. The deposit cancellation event can occur only after a counter deposit is completed successfully.

The transaction fee criteria are shown in Table 5. An interbank ATM withdrawal includes two types of transaction fees, which apply to transactions from the same city as the issuer and transactions from a different city. For an interbank counter deposit, three types of transaction fees exist according to the transaction amount *A*. We implement our approach on fine-grained modules, such as the modules of interbank ATM withdrawal, counter deposit and deposit cancellation.

**Table 5:** Transaction fee criteria.

Interbank transaction	Condition	Transaction fee
interbank ATM withdrawal	from the same city	2
interbank ATM withdrawal	from a different city	2 + 0.01 * *A*
interbank counter deposit	0 < *A* ≤ 3000	3
interbank counter deposit	3000 < *A* < 50000	0.001 * *A*
interbank counter deposit	50000 ≤ *A* ≤ 200000	50

#### Metamorphic relations of interbank ATM withdrawal

For an interbank ATM withdrawal event, the input triggering the event is a 5-tuple (*N*, *A*, *C*_*a*_, *C*_*i*_, *B*_0_), where *N* refers to the card number, *A* refers to the transaction amount, *C*_*a*_ and *C*_*i*_, respectively, refer to the city code of the acquirer and the city code of the issuer, and *B*_0_ refers to the initial balance of card number *N*. Moreover, *C*_*a*_ = *C*_*i*_ indicates that the transaction received by the acquirer is from the same city as the issuer, whereas *C*_*a*_ ≠ *C*_*i*_ indicates that the transaction comes from a different city. The output of an interbank ATM withdrawal is a 3-tuple (*R*, *F*, *B*), where *R*, *F* and *B* represent the response code, transaction fee and balance after the transaction. Note that in this case study, the initial balance *B*_0_ is usually sufficient unless stated otherwise.

1. **MR based on a fixed single-event sequence**. For the fixed single-event sequence ATM withdrawal, if the source input sequence is denoted as *I*_*s*_ = ATM withdrawal(*N*, *A*, *C*_*a*_, *C*_*i*_, *B*_0_), then we can construct the follow-up input sequence $I_{f}=\text{ATM}\;\text{withdrawal}\left(N^{\prime },K\cdot A,C_{a}^{\prime },C_{i}^{\prime },K\cdot B_{0}\right)$ by using another card number *N*′ instead of card number *N*, multiplying the transaction amount *A* and the initial balance *B*_0_ by a positive integer *K*, and changing the city code of acquirer *C*_*a*_ and the city code of issuer *C*_*i*_ to $C_{a}^{{}^{\prime }}$ and $C_{i}^{{}^{\prime }}$, respectively. Suppose the source and follow-up output sequences are described as *O*_*s*_ = (*R*_*s*_, *F*_*s*_, *B*_*s*_) and *O*_*f*_ = (*R*_*f*_, *F*_*f*_, *B*_*f*_), where *R*_*s*_ and *R*_*f*_, *F*_*s*_ and *F*_*f*_, and *B*_*s*_ and *B*_*f*_, respectively, represent the source and follow-up response codes, transaction fees and balances. Thus, we can design MR1.1-MR1.3 as follows.
  **MR1.1:** If both the source and follow-up test cases are transactions from the same city as the issuer, that is, *C*_*a*_ = *C*_*i*_ and $C_{a}^{{}^{\prime }}=C_{i}^{{}^{\prime }}$, the source and follow-up response codes and transaction fees should be identical, and the source balance *B*_*s*_ and the follow-up balance *B*_*f*_ should satisfy the relation *B*_*f*_ = *K* ⋅ *B*_*s*_ + 2(*K* − 1).
  **MR1.2:** If both the source and follow-up test cases are transactions from cities different from that of the issuer, that is, *C*_*a*_ ≠ *C*_*i*_ and $C_{a}^{{}^{\prime }}\neq C_{i}^{{}^{\prime }}$, the source and follow-up response codes should be the same, and the source and follow-up transaction fees (*F*_*s*_ and *F*_*f*_) and balances (*B*_*s*_ and *B*_*f*_) should satisfy the relations *F*_*f*_ = *F*_*s*_ + 0.01(*K* − 1) ⋅ *A* and *B*_*f*_ = *K* ⋅ *B*_*s*_ + 2(*K* − 1).
  **MR1.3:** If the source test case is a transaction from the same city as the issuer, and the follow-up test case is a transaction from a different city, that is, *C*_*a*_ = *C*_*i*_ and $C_{a}^{{}^{\prime }}\neq C_{i}^{{}^{\prime }}$, the source and follow-up response codes should be the same, and the source and follow-up transaction fees (*F*_*s*_ and *F*_*f*_) and balances (*B*_*s*_ and *B*_*f*_) should satisfy the relations *F*_*f*_ = *F*_*s*_ + 0.01*K* ⋅ *A* and *B*_*f*_ = *K* ⋅ *B*_*s*_ + 2(*K* − 1)− 0.01*K* ⋅ *A*.
  **MR1.4:** Similarly, based on the source input sequence *I*_*s*_ = ATM withdrawal(*N*, *A*, *C*_*a*_, *C*_*i*_, *B*_0_), where the relation *C*_*a*_ ≠ *C*_*i*_ means the transaction is from a different city from the issuer, we can construct the follow-up input sequence *I*_*f*_ = ATM withdrawal(*N*, *A* + *C*, *C*_*a*_, *C*_*i*_, *B*_0_ + 2*C*) by increasing the values of the transaction amount *A* and the initial balance *B*_0_ by positive integers *C* (a multiple of 50) and 2*C*. Thus, the source and follow-up response codes should be the same, and the source and follow-up transaction fees (*F*_*s*_ and *F*_*f*_) and balances (*B*_*s*_ and *B*_*f*_) from the source and follow-up output sequences should satisfy the relations *F*_*f*_ = *F*_*s*_ + 0.01*C* and *B*_*f*_ = *B*_*s*_ + 0.99*C*.
  **MR1.5:** Given the source input sequence *I*_*s*_ = ATM withdrawal(*N*, *A*, *C*_*a*_, *C*_*i*_, *B*_0_), we can construct the follow-up input sequence $I_{f}=\text{ATM}\;\text{withdrawal}(N^{\prime },A+K,C_{a}^{\prime },C_{i}^{\prime },B_{0}+C)$ by changing the card number *N* to another card number *N*′, adding a positive integer *K* (a multiple of 50) and a constant *C* to the transaction amount *A* and the initial balance *B*_0_, respectively, and changing the city code of acquirer *C*_*a*_ and the city code of issuer *C*_*i*_ to $C_{a}^{{}^{\prime }}$ and $C_{i}^{{}^{\prime }}$, respectively, where *C*_*a*_ = *C*_*i*_ and $C_{a}^{{}^{\prime }}\neq C_{i}^{{}^{\prime }}$. If the initial balance *B*_0_ of the source input sequence is insufficient and the balance *B*_0_ + *C* of the follow-up input sequence is sufficient, the source response code *R*_*s*_ and the follow-up response code *R*_*f*_ should be different.
2. **MR based on a fixed multi-event sequence**. Suppose the source and follow-up test cases are, respectively, represented as (*E*, *I*_*s*_) and (*E*, *I*_*f*_), where *E* = 〈ATM withdrawal, ATM withdrawal〉 denotes a fixed multi-event sequence of sequentially withdrawing cash twice from the same card. If the source input sequence is denoted as $I_{s}=\langle \text{ATM}\;\text{withdrawal}\left(N,A_{1},C_{a},C_{i},B_{0}\right),\text{ATM}\;\text{withdrawal}\left(N,A_{2},C_{a},C_{i},B_{s}^{1}\right)\rangle$, where $B_{s}^{1}$ represents the new balance after executing the first ATM withdrawal event in the source event sequence, the follow-up input sequence $I_{f}=\langle \text{ATM}\;\text{withdrawal}\left(N^{\prime },K\cdot A_{1},C_{a}^{\prime },C_{i}^{\prime },K\cdot B\right),\text{ATM}\;\text{withdrawal}\left(N^{\prime },K\cdot A_{2},C_{a}^{\prime },C_{i}^{\prime },B_{f}^{1}\right)\rangle$ can be constructed by changing the card number *N* to card number *N*′, multiplying the first withdrawal amount *A*_1_, the second withdrawal amount *A*_2_ and the initial balance *B*_0_ by a positive integer *K*, and changing the city code of acquirer *C*_*a*_ and the city code of issuer *C*_*i*_ to $C_{a}^{{}^{\prime }}$ and $C_{i}^{{}^{\prime }}$, where $B_{f}^{1}$ represents the new balance after executing the first ATM withdrawal event in the follow-up event sequence. Supposing the corresponding output sequences can be denoted as $O_{s}=\langle \left(R_{s}^{1},F_{s}^{1},B_{s}^{1}\right),\left(R_{s}^{2},F_{s}^{1},B_{s}^{2}\right)\rangle$), and $O_{f}=\langle \left(R_{f}^{1},F_{f}^{1},B_{f}^{1}\right),\left(R_{f}^{2},F_{f}^{2},B_{f}^{2}\right)\rangle$, where the superscripts ‘1’ and ‘2’, respectively, refer to the first and second events in the source and follow-up output sequences, the total transaction fee *F*_*s*_ and the final balance *B*_*s*_ in the source output sequence can be calculated using the formulas $F_{s}=F_{s}^{1}+F_{s}^{2}$ and $B_{s}=B_{s}^{2}$, and those in the follow-up output sequence can be calculated via the formulas $F_{f}=F_{f}^{1}+F_{f}^{2}$ and $B_{f}=B_{f}^{2}$. Then, we can obtain metamorphic relations MR1.6-MR1.8.
  **MR1.6:** If the transactions in the source and follow-up input sequences are from the same city, that is, *C*_*a*_ = *C*_*i*_ and $C_{a}^{{}^{\prime }}=C_{i}^{{}^{\prime }}$, the response codes and total transaction fees (*F*_*s*_ and *F*_*f*_) from the source and follow-up output sequences should be the same, and the source and follow-up final balances (*B*_*s*_ and *B*_*f*_) should satisfy the relation *B*_*f*_ = *K* ⋅ *B*_*s*_ + 4(*K* − 1).
  **MR1.7:** If the transactions in the source and follow-up input sequences are from different cities, that is, *C*_*a*_ ≠ *C*_*i*_ and $C_{a}^{{}^{\prime }}\neq C_{i}^{{}^{\prime }}$, the source and follow-up response codes should be the same, and the total transaction fees (*F*_*s*_ and *F*_*f*_) and final balances (*B*_*s*_ and *B*_*f*_) from the source and follow-up output sequences should satisfy the relations *F*_*f*_ = *F*_*s*_ + 0.01(*K* − 1)(*A*_1_ + *A*_2_) and *B*_*f*_ = *K* ⋅ *B*_*s*_ + 4(*K* − 1).
  **MR1.8:** If the source transaction is from the same city and the follow-up transaction is from a different city, that is, *C*_*a*_ = *C*_*i*_ and $C_{a}^{{}^{\prime }}\neq C_{i}^{{}^{\prime }}$, the source and follow-up response codes should be the same, and the total transaction fees (*F*_*s*_ and *F*_*f*_) and final balances (*B*_*s*_ and *B*_*f*_) from the source and follow-up output sequences should satisfy the relations *F*_*f*_ = *F*_*s*_ + 0.01*K*(*A*_1_ + *A*_2_) and *B*_*f*_ = *K* ⋅ *B*_*s*_ + 4(*K* − 1) − 0.01*K*(*A*_1_ + *A*_2_).
  **MR1.9:** If the follow-up input sequence $I_{f}=\langle \text{ATM}\;\text{withdrawal}\left(N,A_{2},C_{a},C_{i},B_{0}\right),\text{ATM}\;\text{withdrawal}\left(N,A_{1},C_{a},C_{i},B_{f}^{1}\right)\rangle$ is constructed by permuting the order of the two transaction amounts *A*_1_ and *A*_2_ of the source input sequence, we can obtain the same source and follow-up response codes, total transaction fees and final balances.
3. **MR based on varied event sequences**. If we sequentially withdraw cash *A*_1_ and *A*_2_ from the same card, the new balance should be related to that calculated by withdrawing cash *A*_1_ + *A*_2_ once. Therefore, we suppose *E*_*s*_ = 〈ATM withdrawal, ATM withdrawal〉 is the source event sequence that sequentially withdraws cash twice; the source input sequence is given as $I_{s}=\langle \text{ATM}\;\text{withdrawal}\left(N,A_{1},C_{a},C_{i},B_{0}\right),\text{ATM}\;\text{withdrawal}\left(N,A_{2},C_{a},C_{i},B_{s}^{1}\right)\rangle$. Thus, we can construct the follow-up test case (*E*_*f*_, *I*_*f*_), where the follow-up event sequence *E*_*f*_ = ATM withdrawal is constructed by deleting an ATM withdrawal event from the source event sequence. The follow-up input sequence $I_{f}=\text{ATM}\;\text{withdrawal}\left(N^{\prime },A_{1}+A_{2},C_{a}^{\prime },C_{i}^{\prime },B_{0}\right)$ is constructed by withdrawing cash *A*_1_ + *A*_2_ once, changing the card number from *N* to *N*′, changing the city code of the acquirer from *C*_*a*_ to $C_{a}^{{}^{\prime }}$ and changing the city code of the issuer from *C*_*i*_ to $C_{i}^{{}^{\prime }}$. If the source and follow-up output sequences are, respectively, denoted as $O_{s}=\langle \left(R_{s}^{1},F_{s}^{1},B_{s}^{1}\right),\left(R_{s}^{2},F_{s}^{2},B_{s}^{2}\right)\rangle$ and *O*_*f*_ = 〈(*R*_*f*_, *F*_*f*_, *B*_*f*_)〉, the total transaction fee *F*_*s*_ and the final balance *B*_*s*_ in the source output sequence can be calculated using the formulas $F_{s}=F_{s}^{1}+F_{s}^{2}$ and $B_{s}=B_{s}^{2}$. Then, we can design the following MRs.
  **MR1.10:** If the source and follow-up test cases are transactions from the same city as the issuer, that is, *C*_*a*_ = *C*_*i*_ and $C_{a}^{{}^{\prime }}=C_{i}^{{}^{\prime }}$, the source and follow-up response codes should be the same, and the total transaction fees (*F*_*s*_ and *F*_*f*_) and final balances (*B*_*s*_ and *B*_*f*_) from the source and follow-up output sequences should satisfy the relations *F*_*f*_ = *F*_*s*_ − 2 and *B*_*f*_ = *B*_*s*_ + 2.
  **MR1.11:** The difference of this MR from *MR*1.10 is that the source test case is a transaction from the same city as the issuer but the follow-up test case is a transaction from a different city, that is, *C*_*a*_ = *C*_*i*_ and $C_{a}^{{}^{\prime }}\neq C_{i}^{{}^{\prime }}$. Thus, the source and follow-up response codes should be the same, and the total transaction fees (*F*_*s*_ and *F*_*f*_) and final balances (*B*_*s*_ and *B*_*f*_) from the source and follow-up output sequences should satisfy the relations *F*_*f*_ = *F*_*s*_ − 2 + 0.01(*A*_1_ + *A*_2_) and *B*_*f*_ = *B*_*s*_ + 2 − 0.01(*A*_1_ + *A*_2_).

#### Metamorphic relations of interbank counter deposit

For the interbank counter deposit event, the input is a 4-tuple (*N*, *S*, *A*, *B*_0_), where *N*, *S*, *A* and *B*_0_ represent the card number, sequence number, transaction amount and the initial balance of the card, respectively. The output is identical to the output sequence of an interbank ATM withdrawal, namely, response code *R*, transaction fee *F* and new balance *B*.

1. **MR based on a fixed single-event sequence**. We suppose that the source test case is (counter deposit, *I*_*s*_) and that the follow-up test case is (counter deposit, *I*_*f*_). Given the source input sequence *I*_*s*_ = counter deposit(*N*, *S*, *A*, *B*_0_), we can construct the follow-up input sequence *I*_*f*_ = counter deposit(*N*, *S*′, *K* · *A*, *K* · *B*_0_) by multiplying the transaction amount *A* and the initial balance *B*_0_ by a positive integer *K* and changing the sequence number of the counter deposit from *S* to *S*′. The source and follow-up output sequences are denoted as *O*_*s*_ = (*R*_*s*_, *F*_*s*_, *B*_*s*_) and *O*_*f*_ = (*R*_*f*_, *F*_*f*_, *B*_*f*_), where *R*_*s*_ and *R*_*f*_, *F*_*s*_ and *F*_*f*_, and *B*_*s*_ and *B*_*f*_, respectively, represent the source and follow-up response codes, transaction fees and balances. Then, we can design MR2.1-MR2.3.
  **MR2.1:** If both the source and follow-up transaction amounts *A* and *K* ⋅ *A* are within the range [50000, 200000], the source and follow-up output sequences should have the same response code and transaction fee, and satisfy the relation between the source and follow-up balances *B*_*f*_ = *K* ⋅ *B*_*s*_ + 50(*K* − 1).
  **MR2.2:** If both the source and follow-up transaction amounts *A* and *K* ⋅ *A* are within the range (3000, 50000), the outputs of the follow-up test case should be *K* times those of the source test case, with the exception of the same response code, that is, the follow-up transaction fee *F*_*f*_ = *K* ⋅ *F*_*s*_ and the follow-up balance *B*_*f*_ = *K* ⋅ *B*_*s*_.
  **MR2.3:** If the source transaction amount *A* is within the range (3000, 50000) and the follow-up transaction amount *K* ⋅ *A* is within the range [50000, 200000], the source and follow-up response codes should be the same, and the source and follow-up transaction fees (*F*_*s*_ and *F*_*f*_) and balances (*B*_*s*_ and *B*_*f*_) should satisfy the relations *F*_*f*_ = *F*_*s*_ + 50 − 0.001*A* and *B*_*f*_ = *K* ⋅ *B*_*s*_ + 0.001*K* ⋅ *A* − 50.
  Given a source input sequence *I*_*s*_ = counter deposit(*N*, *S*, *A*, *B*_0_), we can construct a follow-up input sequence *I*_*f*_ = counter deposit(*N*, *S*′, *A* + *K*, *B*_0_ + *C*) by increasing the values of the transaction amount *A* and the initial balance *B*_0_ by constants *K* and *C* and changing the sequence number from *S* to *S*′. Then, we can construct the following MRs.
  **MR2.4:** If both the source and follow-up transaction amounts *A* and *A* + *K* are within the range (0, 3000], we can obtain the same source and follow-up response codes and transaction fees, and the output relation between the source and follow-up balances *B*_*f*_ = *B*_*s*_ + *K* + *C*.
  **MR2.5:** If the source transaction amount *A* is within the range (0, 3000] and the follow-up transaction amount *A* + *k* is within the range [50000, 200000], the source and follow-up output sequences should have output relations with the follow-up transaction fee *F*_*f*_ = *F*_*s*_ + 47 and the follow-up balance *B*_*f*_ = *B*_*s*_ + *C* + *K* − 47 and the same response code.
2. **MR based on a fixed multi-event sequence**. Suppose the source and follow-up test cases are denoted as (*E*, *I*_*s*_) and (*E*, *I*_*f*_). The fixed multi-event sequence *E* = 〈counter deposit, counter deposit〉 represents sequentially depositing cash twice, and the source input sequence $I_{s}=\langle \text{counter}\;\text{deposit}\left(N,S_{1},A_{1},B_{0}\right),\text{counter}\;\text{deposit}\left(N,S_{2},A_{2},B_{s}^{1}\right)\rangle$ denotes depositing cash *A*_1_ and *A*_2_ onto the card number *N* with the initial balance *B*_0_, where $B_{s}^{1}$ represents the new balance after executing the first counter deposit event. Assuming the source and follow-up output sequences are expressed as $O_{s}=\langle \left(R_{s}^{1},F_{s}^{1},B_{s}^{1}\right),\left(R_{s}^{2},F_{s}^{2},B_{s}^{2}\right)\rangle$ and $O_{f}=\langle \left(R_{f}^{1},F_{f}^{1},B_{f}^{1}\right),\left(R_{f}^{2},F_{f}^{2},B_{f}^{2}\right)\rangle$, the total transaction fee *F*_*s*_ and the final balance *B*_*s*_ from the source output sequence can be obtained using the formulas $F_{s}=F_{s}^{1}+F_{s}^{2}$ and $B_{s}=B_{s}^{2}$, and those in the follow-up output sequence can be obtained using the formulas $F_{f}=F_{f}^{1}+F_{f}^{2}$ and $B_{f}=B_{f}^{2}$.
  **MR2.6:** If the follow-up input sequence $I_{f}=\langle \text{counter}\;\text{deposit}(N,S_{1},K\cdot A_{1},B_{0}+C),\text{counter}\;\text{deposit}\left(N,S_{2},K\cdot A_{2},B_{f}^{1}\right)\rangle$ is constructed by multiplying the transaction amounts *A*_1_ and *A*_2_ by a positive integer *K* and adding a positive constant *C* to the initial balance *B*_0_, where *A*_1_ ∈ (0, 3000], *A*_2_ ∈ [50000, 200000], *K* ⋅ *A*_1_ ∈ (3000, 50000), and *K* ⋅ *A*_2_ ∈ [50000, 200000], we can obtain the same source and follow-up response codes, the relations with the follow-up total transaction fee *F*_*f*_ = *F*_*s*_ + 0.001*K* ⋅ *A*_1_ − 3 and with the follow-up final balance *B*_*f*_ = *B*_*s*_ + (*K* − 1) ⋅ (*A*_1_ + *A*_2_) − 0.001*K* ⋅ *A*_1_ + *C* + 3.
  **MR2.7:** If the follow-up input sequence $I_{f}=\langle \text{counter}\;\text{deposit}(N,S_{1},A_{1}+C,B_{0}+C),\text{counter}\;\text{deposit}\left(N,S_{2},A_{2}+C,B_{f}^{1}\right)\rangle$ is constructed by adding a constant *C* to the transaction amounts *A*_1_ and *A*_2_ and initial balance *B*_0_, where *A*_1_, *A*_2_, *A*_1_ + *C* and *A*_2_ + *C* are all within the range (3000, 50000), we can obtain the same source and follow-up response codes, the relations between the source and follow-up total transaction fees *F*_*f*_ = *F*_*s*_ + 0.002*C* and between the source and follow-up final balances *B*_*f*_ = *B*_*s*_ + 2.998*C*.
  **MR2.8:** If the follow-up input sequence $I_{f}=\langle \text{counter}\;\text{deposit}\left(N,S_{1},A_{2},B_{0}\right),\text{counter}\;\text{deposit}\left(N,S_{2},A_{1},B_{f}^{1}\right)\rangle$ is constructed by permuting the order of two transaction amounts *A*_1_ and *A*_2_ of the source input sequence, where *A*_1_ and *A*_2_ are within the range [50000, 200000], the source and follow-up output sequences should have the same response code, total transaction fee and final balance.
3. **MR based on varied event sequences**. Suppose (*E*_*s*_, *I*_*s*_) is the source test case, where the source event sequence and input sequence are denoted as *E*_*s*_ = 〈counter deposit, counter deposit〉 and $I_{s}=\langle \text{counter}\;\text{deposit}\left(N,S_{1},A_{1},B_{0}\right),\text{counter}\;\text{deposit}\left(N,S_{2},A_{2},B_{s}^{1}\right)\rangle$. We can construct the follow-up test case (*E*_*f*_, *I*_*f*_) of depositing cash *A*_1_ + *A*_2_ once, where the follow-up event sequence *E*_*f*_ = counter deposit is constructed by deleting a counter deposit event from the source event sequence, and the follow-up input sequence is denoted as *I*_*f*_ = counter deposit(*N*, *S*_3_, *A*_1_ + *A*_2_, *B*_0_). Thus, the source and follow-up output sequences can be represented as $O_{s}=\langle \left(R_{s}^{1},F_{s}^{1},B_{s}^{1}\right),\left(R_{s}^{2},F_{s}^{2},B_{s}^{2}\right)\rangle$ and *O*_*f*_ = (*R*_*f*_, *F*_*f*_, *B*_*f*_), respectively. Then, the source total transaction fee *F*_*s*_ and the source final balance *B*_*s*_ from the source output sequence can be obtained using the formulas $F_{s}=F_{s}^{1}+F_{s}^{2}$ and $B_{s}=B_{s}^{2}$. We can then obtain the metamorphic relations MR2.9-MR2.11.
  **MR2.9:** If the source transaction amounts *A*_1_ and *A*_2_ and the follow-up transaction amount *A*_1_ + *A*_2_ are all within the range [50000, 200000], the source and follow-up output sequences should satisfy the relations *F*_*f*_ = *F*_*s*_ − 50 and *B*_*f*_ = *B*_*s*_ + 50, except that they have the same response code.
  **MR2.10:** If the source transaction amounts *A*_1_ and *A*_2_ and the follow-up transaction amount *A*_1_ + *A*_2_ are all within the range (3000, 50000), the source and follow-up output sequences should have the same response code, total transaction fee and final balance.
  **MR2.11:** Supposing the source transaction amounts *A*_1_ ∈ (0, 3000] and *A*_2_ ∈ (3000, 50000) and the follow-up transaction amount *A*_1_ + *A*_2_ ∈ [50000, 200000], the source and follow-up output sequences should satisfy the relations *F*_*f*_ = *F*_*s*_ + 47 − 0.001*A*_2_ and *B*_*f*_ = *B*_*s*_ + 0.001*A*_2_ − 47, and have the same response code.

#### Metamorphic relations of deposit cancellation

We investigate an event sequence that executes a deposit cancellation after sequentially executing two counter deposits. The aim of this test is to check whether the deposit cancellation event can correctly cancel the deposit transaction. Suppose the source test case is denoted as (*E*_*s*_, *I*_*s*_), where *E*_*s*_ is 〈counter deposit, counter deposit, deposit cancellation〉 and *I*_*s*_ is 〈counter deposit (*N*, *S*_1_, *A*_1_, *B*_0_), counter deposit(*N*, *S*_2_, *A*_2_, $B_{s}^{1}$), deposit cancellation(*S*_2_, *A*_2_)〉. The input of deposit cancellation (*S*_2_, *A*_2_) is derived from the input of the second counter deposit event, which means that the second transaction is withdrawn. Thus, if the source output sequence is denoted as $O_{s}=\langle \left(R_{s}^{1},F_{s}^{1},B_{s}^{1}\right),\left(R_{s}^{2},F_{s}^{2},B_{s}^{2}\right),\left(R_{s}^{3},B_{s}^{3}\right)\rangle$, where $R_{s}^{3}$ and $B_{s}^{3}$, respectively, denote the response code and the new balance after deposit cancellation, the total transaction fee and the final balance should be the unrepealed transaction fee $F_{s}^{1}$ and the balance $B_{s}^{3}$ after all event executions. According to the rules of banks, a deposit cancellation cannot be executed until a counter deposit transaction is successfully executed. Therefore, we cannot randomly change the order of the counter deposit and deposit cancellation.

1. **MR based on a fixed multi-event sequence**. If the follow-up test case has the same event sequence as the source test case, the follow-up output sequence can be denoted as $O_{f}=\langle \left(R_{f}^{1},F_{f}^{1},B_{f}^{1}\right),\left(R_{f}^{2},F_{f}^{2},B_{f}^{2}\right),\left(R_{f}^{3},B_{f}^{3}\right)\rangle$, where $R_{f}^{3}$ and $B_{f}^{3}$ denote the response code and the final balance after deposit cancellation. Then, we can design the following MRs.
  **MR3.1:** If the follow-up input sequence *I*_*f*_ = 〈counter deposit(*N*, *S*_1_, *A*_1_, *B*_0_), counter deposit(*N*, *S*_2_, *A*_2_, $B_{f}^{1}$), deposit cancellation(*S*_1_, *A*_1_)〉 is constructed by changing the input of the deposit cancellation event from the input of the second event (*S*_2_, *A*_2_) to the input of the first event (*S*_1_, *A*_1_), where the deposit amounts *A*_1_ ∈ (0, 3000] and *A*_2_ ∈ (3000, 50000), then we can obtain the same source and follow-up response codes, the relation between the source and follow-up unrepealed transaction fees, $F_{f}^{2}=F_{s}^{1}+0.001\cdot A_{2}-3$, and the relation between the source and follow-up final balances, $B_{f}^{3}=B_{s}^{3}+0.999\cdot A_{2}-A_{1}+3$.
  **MR3.2:** If the follow-up input sequence *I*_*f*_ = 〈counter deposit(*N*, *S*_1_, *K* ⋅ *A*_1_, *K* ⋅ *B*_0_), counter deposit(*N*, *S*_2_, *K* · *A*_2_, $B_{f}^{1}$), deposit cancellation(*S*_2_, *K* ⋅ *A*_2_)〉 is constructed by multiplying the deposit amounts *A*_1_ and *A*_2_ and the initial balance *B*_0_ by a positive integer *K*, where the source and follow-up deposit amounts *A*_1_ ∈ (0, 3000], *A*_2_ ∈ (3000, 50000), *K* ⋅ *A*_1_ ∈ (3000, 50000) and *K* ⋅ *A*_2_ ∈ [50000, 200000], then we can obtain the same source and follow-up response codes, the output relations for the follow-up unrepealed transaction fee, $F_{f}^{1}=F_{s}^{1}+0.001K\cdot A_{1}-3$, and the follow-up final balance, $B_{f}^{3}=K\cdot B_{s}^{3}-0.001K\cdot A_{1}+3K$.
  **MR3.3:** If the follow-up input sequence *I*_*f*_ = 〈counter deposit(*N*, *S*_1_, *A*_1_ + *C*, *B*_0_ + *C*), counter deposit(*N*, *S*_2_, *A*_2_ + *C*, $B_{f}^{1}$), deposit cancellation(*S*_2_, *A*_2_ + *C*)〉 is constructed by increasing the values of the deposit amounts *A*_1_ and *A*_2_ and the initial balance *B*_0_ by a constant *C*, where the deposit amounts *A*_1_, *A*_2_, *A*_1_ + *C* and *A*_2_ + *C* are all within (3000, 50000), we can obtain the same source and follow-up response codes, the relations for the follow-up unrepealed transaction fee, $F_{f}^{1}=F_{s}^{1}+0.001C$, and the follow-up final balance, $B_{f}^{3}=B_{s}^{3}+1.999C$.
  **MR3.4:** This MR is similar to MR3.3, except for the follow-up input sequence *I*_*f*_ = 〈counter deposit(*N*, *S*_2_, *A*_2_ + *C*, *B*_0_ + *C*), counter deposit(*N*, *S*_1_, *A*_1_ + *C*, $B_{f}^{1}$), deposit cancellation(*S*_2_, *A*_2_ + *C*)〉. The difference of this MR is that the source and follow-up input sequences have different transaction amount ranges, that is, *A*_1_ ∈ (0, 3000], *A*_2_ ∈ (0, 3000], *A*_1_ + *C* ∈ (3000, 50000), and *A*_2_ + *C* ∈ (3000, 50000). Thus, we can obtain the same source and follow-up response codes, the relation between the source and follow-up unrepealed transaction fees, $F_{f}^{1}=F_{s}^{1}+0.001\left(A_{1}+C\right)-3$, and the relation between the source and follow-up final balances, $B_{f}^{3}=B_{s}^{3}+0.999C-0.001A_{1}+3$.
2. **MR based on varied event sequences**. Based on the source event sequence *E*_*s*_ = 〈counter deposit, counter deposit, deposit cancellation〉 and the input sequence *I*_*s*_, we can construct the following MRs.
  **MR3.5:** The follow-up event sequence *E*_*f*_ = 〈counter deposit, counter deposit〉 and its corresponding input sequence *I*_*f*_ = 〈counter deposit(*N*, *S*_1_, *A*_1_, *B*_0_), counter deposit(*N*, *S*_2_, *A*_2_, $B_{f}^{1}$)〉 can be constructed by deleting the deposit cancellation event from the source event sequence, where the deposit amounts *A*_1_, *A*_2_ ∈ [50000, 200000]. Thus, the follow-up output sequence can be denoted as $O_{f}=\langle \left(R_{f}^{1},F_{f}^{1},B_{f}^{1}\right),\left(R_{f}^{2},F_{f}^{2},B_{f}^{2}\right)\rangle$. Then, the source and follow-up output sequences should have the same response code, and satisfy the follow-up transaction fee relation $F_{f}^{1}+F_{f}^{2}=F_{s}^{1}+50$ and the follow-up final balance relation $B_{f}^{2}=B_{s}^{3}+A_{2}-50$.
  **MR3.6:** Compared with *MR*3.5, the follow-up event sequence of this MR *E*_*f*_ = counter deposit is constructed by deleting a counter deposit event and its corresponding deposit cancellation event from the source event sequence. The corresponding follow-up input sequence and output sequence are represented as *I*_*f*_ = counter deposit(*N*, *S*_1_, *A*_1_, *B*_0_) and *O*_*f*_ = (*R*_*f*_, *F*_*f*_, *B*_*f*_). In this case, the source and follow-up output sequences should have the same response code, total transaction fee and final balance, that is, $R_{f}=R_{s}^{3}$, $F_{f}=F_{s}^{1}$ and $B_{f}=B_{s}^{3}$.

#### Experimental results and analysis

For each MR, we use random testing to generate the source input sequences. Considering the limitations of ATM withdrawal, we generate 50, 200 and 200 valid test groups for each MR from ATM withdrawal, counter deposit and deposit cancellation, respectively. Then, we use mutation analysis to separately generate 65 and 58 mutants for the modules of ATM withdrawal and counter deposit. The event sequence involving two modules of counter deposit and deposit cancellation includes 85 non-equivalent mutants. We execute all test groups, compare their output sequences and evaluate the effectiveness in terms of MS.

Table 6 summarizes the MS of each ATM withdrawal MR for all mutants. MRs based on varied event sequences have higher fault-detection capabilities. *MR*1.11 is the strongest and kills nearly 90% of all mutants, whereas *MR*1.5, based on a fixed single-event sequence, is the weakest and kills only 16.92% of all mutants. For the same type of metamorphic relations, different MRs have different fault-detection capabilities. For instance, *MR*1.3 is more effective than other MRs based on a fixed single-event sequence, *MR*1.8 is more effective than other MRs based on a fixed multi-event sequence, and *MR*1.11 is more effective than *MR*1.10, which is based on varied event sequences. Further analysis reveals that MRs that conduct executions of the source and follow-up test cases in different ways are more likely to reveal faults. In *MR*1.11, the execution of the follow-up test case is performed with a more different input sequence, different event sequence and different execution path than those of the source test case, whereas *MR*1.10 uses only a different input sequence and a different event sequence. For a fixed multi-event sequence, *MR*1.8 includes different input sequences and different execution paths, whereas the other MRs include only different input sequences. The same situation occurs for MRs based on a fixed single-event sequence, except for *MR*1.5. *MR*1.5 is less effective than *MR*1.4 even though it has more different execution paths. Further observation indicates that *MR*1.5 includes only one output parameter, while *MR*1.4 includes three output parameters. *MR*1.5 has a ‘loose’ output relation, which deteriorates the fault-detection effectiveness.

**Table 6:** Mutation scores of MRs for the ATM withdrawal event for all mutants.

MR	MR1.1	MR1.2	MR1.3	MR1.4	MR1.5
MS	60.00%	78.46%	83.08%	49.23%	16.92%
MR1.6	MR1.7	MR1.8	MR1.9	MR1.10	MR1.11
64.62%	78.46%	86.15%	24.62%	52.31%	89.23%

Table 7 shows the MS of each MR for the counter deposit event for all mutants. The MRs derived from different test scenarios have different fault-detection effectiveness. For instance, *MR*2.11, which is based on varied event sequences, is the strongest metamorphic relation and kills 81.03% of all mutants. *MR*2.3, which is based on a fixed single-event sequence, kills 74.14% of all mutants, while *MR*2.8, which is based on a fixed multi-event sequence, kills only 12.07% of all mutants. In addition, the MRs with greater differences in the executions of the SUT have higher fault-detection capabilities. For example, *MR*2.3 kills more mutants than do the other MRs based on a fixed single-event sequence because the execution of its follow-up test case involves more different execution paths and richer input and output relations. *MR*2.6 is more effective than the other MRs based on a fixed multi-event sequence because of more different execution paths and richer input relations. MRs based on varied event sequences are usually more effective. For instance, *MR*2.11 is the most effective of all MRs due to more different event sequences and execution paths. *MR*2.9 and *MR*2.10 are more effective than the other MRs due to more different event sequences, except the abovementioned *MR*2.3, which has more different execution paths.

**Table 7:** Mutation scores of MRs for the counter deposit event for all mutants.

MR	MR2.1	MR2.2	MR2.3	MR2.4	MR2.5
MS	55.17%	62.07%	74.14%	25.86%	46.55%
MR2.6	MR2.7	MR2.8	MR2.9	MR2.10	MR2.11
65.52%	44.83%	12.07%	65.52%	70.69%	81.03%

The same phenomenon exists in Table 8, which shows the results of the event sequence involving counter deposit and deposit cancellation. *MR*3.1-*MR*3.6 have different fault-detection capabilities. *MR*3.2 is the best metamorphic relation, killing 85.88% of all mutants, whereas the worst metamorphic relation, *MR*3.1, kills only 63.53% of all mutants. Furthermore, the best MRs are those that make the executions of the source and follow-up test cases as different as possible. For instance, *MR*3.2 and *MR*3.4 are more effective than the other MRs because they involve more different input sequences and execution paths. Although both *MR*3.5 and *MR*3.6 involve varied event sequences, the executions of their source and follow-up test cases partially go through the same execution path and input sequence. Therefore, *MR*3.5 and *MR*3.6 are less effective than *MR*3.2-*MR*3.4. Moreover, the effectiveness of a metamorphic relation is related to multiple factors.

**Table 8:** Mutation scores of MRs for the deposit cancellation event for all mutants.

MR3.1	MR3.2	MR3.3	MR3.4	MR3.5	MR3.6
63.53%	85.88%	72.94%	76.47%	68.24%	68.24%

We further analyze the experimental results with respect to different types of mutants. Each MR for ATM withdrawal has variable sensitivity to different types of mutants from Table 9. For instance, *MR*1.4 can kill 86.96% of mathematics mutants and 75% of condition mutants, but it cannot kill any off-by-one mutant. *MR*1.3, *MR*1.8 and *MR*1.11 are sensitive to all types of mutants, and their MSs are identical for condition mutants. Among these three MRs, *MR*1.3 has a slightly lower MS than the other MRs for mathematics mutants, and *MR*1.11 has the highest MS of up to 100% for off-by-one mutants.

**Table 9:** Mutation scores of MRs for the ATM withdrawal event for different types of mutants.

	Mathematics	Off-by-one	Condition
MR1.1	39.13%	76.92%	62.50%
MR1.2	73.91%	84.62%	75.00%
MR1.3	73.91%	92.31%	81.25%
MR1.4	86.96%	0.00%	75.00%
MR1.5	21.74%	7.69%	25.00%
MR1.6	47.83%	76.92%	68.75%
MR1.7	82.61%	76.92%	75.00%
MR1.8	82.61%	92.31%	81.25%
MR1.9	47.83%	0.00%	31.25%
MR1.10	47.83%	69.23%	31.25%
MR1.11	82.61%	100.00%	81.25%

Table 10 shows that *MR*2.4, *MR*2.7 and *MR*2.8 are insensitive to off-by-one mutants and cannot kill any off-by-one mutant. However, *MR*2.2 and *MR*2.10 are the most sensitive MRs to off-by-one mutants, with the MSs of 100%. *MR*2.6 and *MR*2.7 are very sensitive to mathematics mutants, with the MSs of 80%. Among all MRs, *MR*2.11 is the strongest MR and is sensitive to all types of mutants, whereas *MR*2.8 is the weakest MR and kills only 35% of mathematics mutants.

**Table 10:** Mutation scores of MRs for the counter deposit event for different types of mutants.

	Mathematics	Off-by-one	Condition
MR2.1	40.00%	63.64%	62.50%
MR2.2	20.00%	100.00%	62.50%
MR2.3	65.00%	81.82%	75.00%
MR2.4	50.00%	0.00%	31.25%
MR2.5	55.00%	18.18%	75.00%
MR2.6	80.00%	36.36%	87.50%
MR2.7	80.00%	0.00%	62.50%
MR2.8	35.00%	0.00%	0.00%
MR2.9	50.00%	81.82%	62.50%
MR2.10	45.00%	100.00%	62.50%
MR2.11	75.00%	81.82%	87.50%

The same situation exists in Table 11. Each MR has different sensitivities to different types of mutants. *MR*3.4 kills 100% of mathematics mutants and 80% of condition mutants, but it kills only 46.67% of off-by-one mutants. *MR*3.2 is sensitive to all types of mutants, with the MSs of 80% or higher. *MR*3.1, *MR*3.3 and *MR*3.4 have the same MS for off-by-one mutants, and *MR*3.1, *MR*3.2 and *MR*3.4 have the same sensitivity to condition mutants.

**Table 11:** Mutation scores of MRs for the deposit cancellation event for different types of mutants.

	Mathematics	Off-by-one	Condition
MR3.1	68.57%	46.67%	80.00%
MR3.2	88.57%	86.67%	80.00%
MR3.3	97.14%	46.67%	70.00%
MR3.4	100.00%	46.67%	80.00%
MR3.5	57.14%	80.00%	70.00%
MR3.6	85.71%	80.00%	20.00%

**Fig 10:**
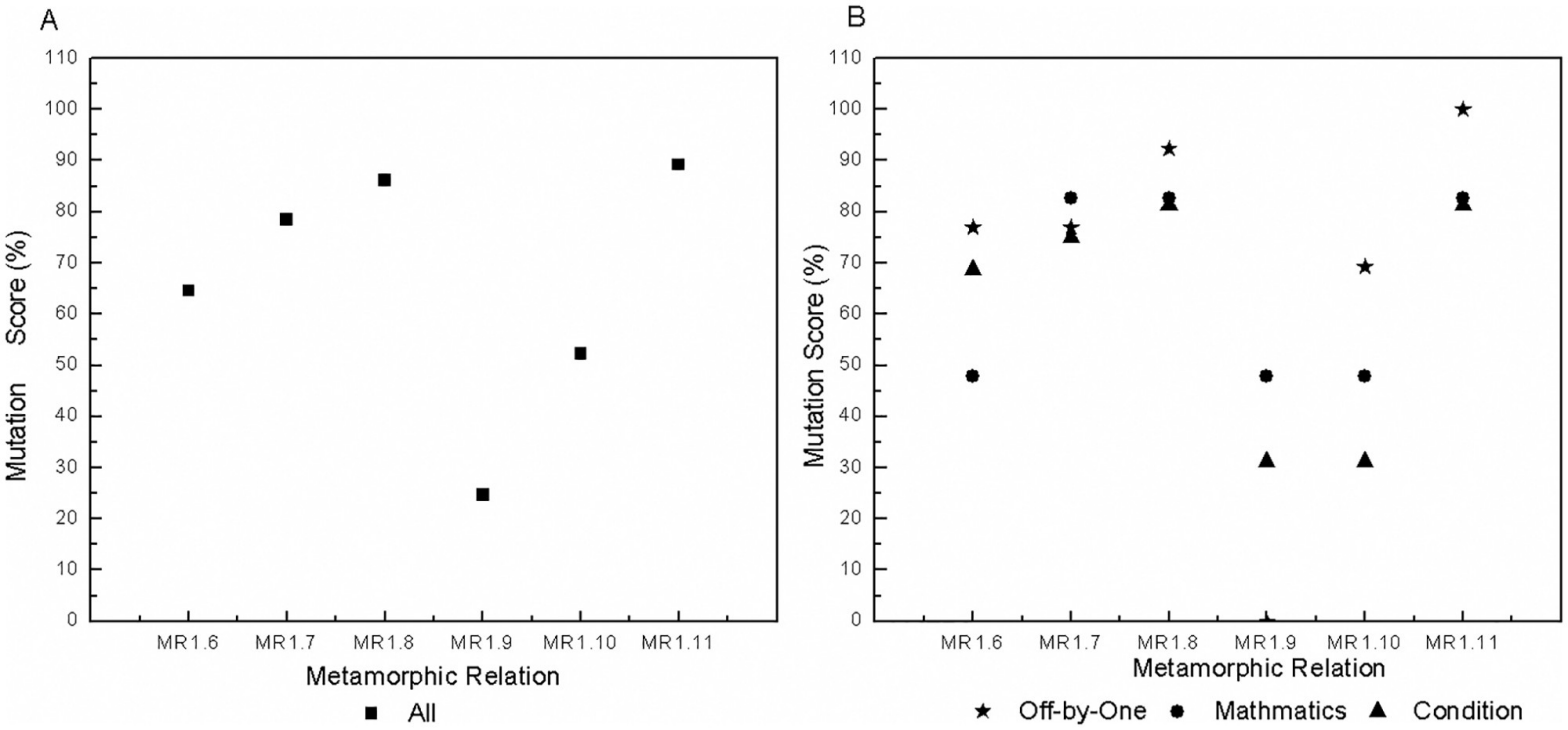
Mutation scores of the MRs based on the same event sequence 〈ATM withdrawal,ATM withdrawal〉. A: Those for all mutants. B: Those for different types of mutants.

**Fig 11:**
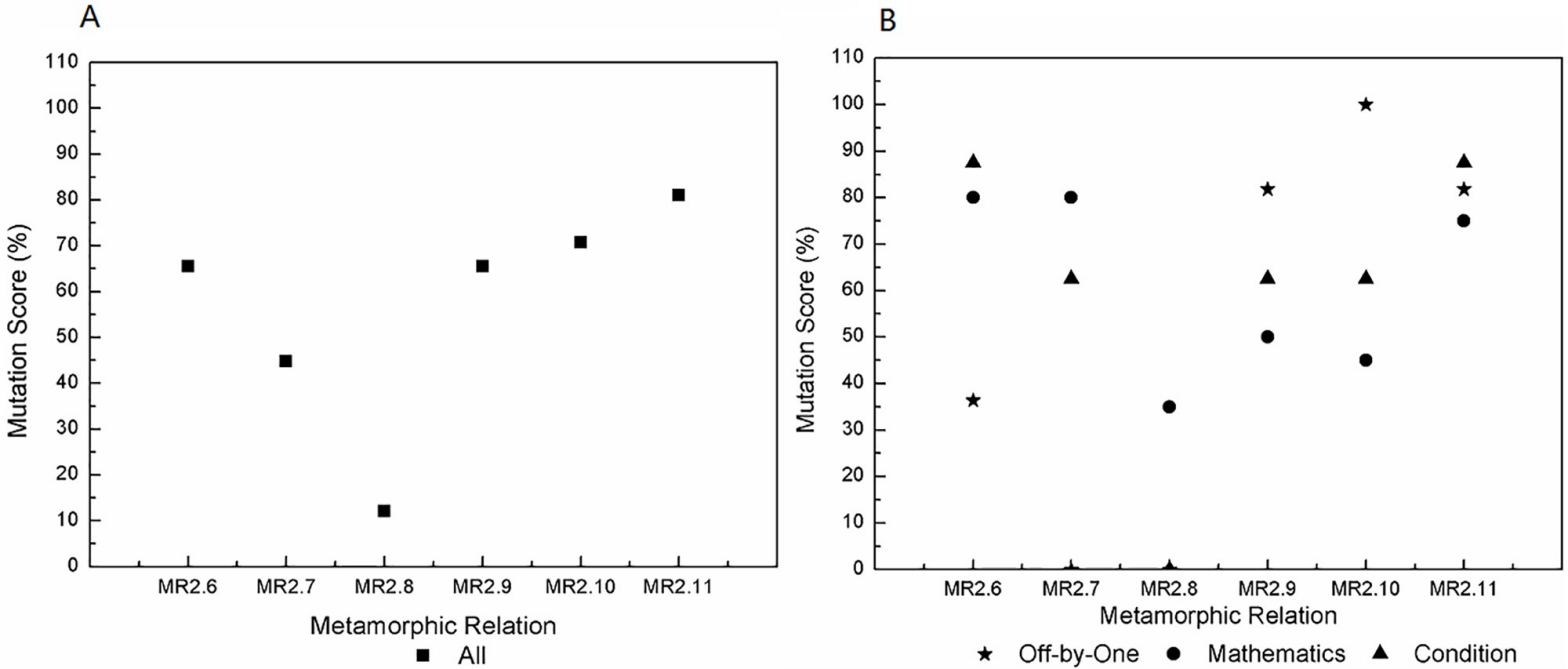
Mutation scores of the MRs based on the same source event sequence 〈counter deposit,counter deposit〉. A: Those for all mutants. B: Those for different types of mutants.

**Fig 12:**
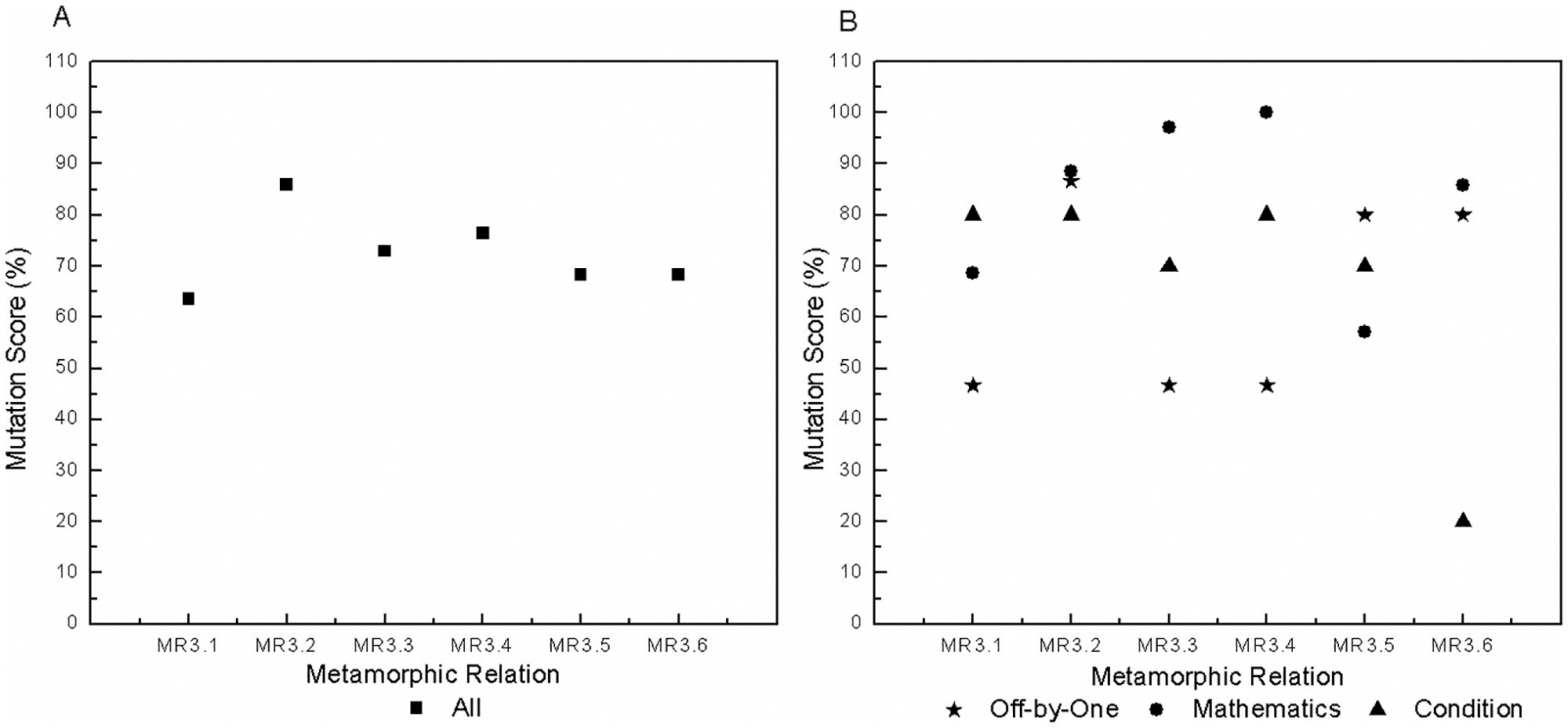
Mutation scores of the MRs based on the same source event sequence 〈counter deposit,counter deposit,deposit cancellation〉. A: Those for all mutants. B: Those for different types of mutants.

For illustration, Figs 10–12 show the MSs of the MRs based on the same source event sequences. Off-by-one mutants are difficult to kill for MRs with the transformation of an addition or a permutation between the source and follow-up input sequences. For instance, *MR*1.9 for ATM withdrawal, *MR*2.7 and *MR*2.8 for counter deposit, and *MR*3.1, *MR*3.3 and *MR*3.4 for deposit cancellation are not sensitive to off-by-one mutants. Furthermore, MRs with identical execution paths (i.e., determination conditions), such as *MR*1.9, *MR*1.10, *MR*2.8 and *MR*3.6, have low fault-detection capabilities for condition mutants. Moreover, MRs with richer input and output relations, such as *MR*1.7, *MR*1.8, *MR*1.11, *MR*2.6, *MR*2.7 and *MR*3.4, are more sensitive to mathematic mutants. MRs with more different event sequences and richer input and output relations, such as *MR*1.11, *MR*2.11 and *MR*3.2, are more effective and sensitive for all types of mutants.

### Case study 3

#### An elastic cloud management system

Cloud computing has been widely applied in the information technology (IT) industry with rich resources and a pay-as-you-go cost model. Cloud computing integrates various computational, storage and network resources into a large pool to benefit a large number of users’ resource demands simultaneously. Based on virtualization techniques, users can request various virtual machines (VMs) and virtual clusters as needed. Users can also release some or all VMs when they do not need as many resources.

Autoscaling is an effective method to ensure the quality of service of users’ applications. Autoscaling can dynamically reallocate resources to enhance application performance or reduce users’ cost when the resource utilization is above or below a preset threshold. For example, a virtual cluster with 10 VMs is created to run a web application on a cloud platform. When the average resource utilization of this virtual cluster (e.g., CPU utilization) exceeds a preset threshold (e.g., 80%) during a fixed observation period, the application performance will decrease. At this moment, this cluster will automatically add one or more VMs according to the predefined autoscaling strategy to improve the application performance. Conversely, one or more VMs can be removed to reduce users’ resource cost when the average resource utilization of the cluster is below a preset threshold.

**Fig 13:**
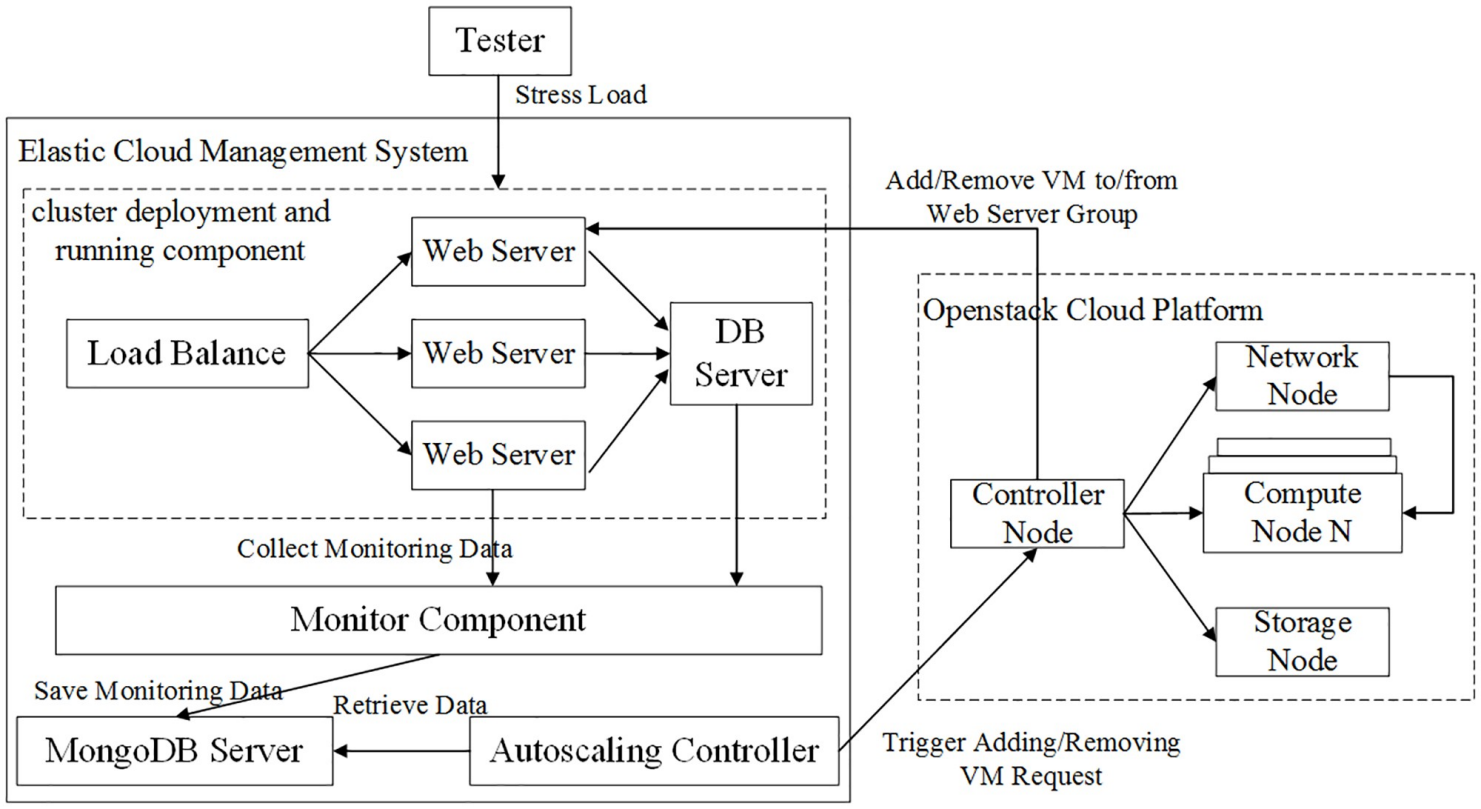
An elastic cloud management system based on an Openstack cloud platform.

**Fig 14:**
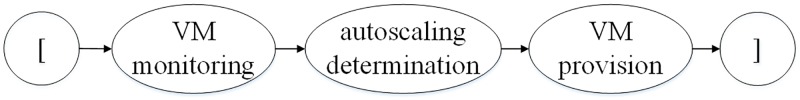
ESG of an elastic cloud management system.

Figs 13 and 14 show an elastic cloud management system and its ESG, respectively. This system manages an Openstack platform composed of 13 physical servers (1 controller node, 1 network node, 1 storage node and 10 compute nodes). A round-robin scheduling strategy is applied to determine on which compute node a VM will be created. The elastic cloud management system includes many components, three of which are related mainly to autoscaling: the cluster deployment and running component, the monitor component and the autoscaling controller. A user submits a request for a three-tier web application cluster to this system, including the number and configuration of the requested VMs and the runtime environment of the web application. The cluster deployment and running component automatically create the VMs and deploy the application on the VMs, which completes the creation of the web application cluster. When the cluster is running, the monitor component collects the real-time resource utilizations of the VMs and periodically saves the data in a MongoDB database. Simultaneously, the autoscaling controller periodically retrieves the data (i.e., resource utilization of the VMs) from the MongoDB database to compute the average resource utilization of the cluster and to determine whether the cluster can increase or decrease the number of VMs according to the autoscaling strategy. If the average resource utilization of the cluster exceeds the predefined upper threshold of the autoscaling strategy, it will trigger the Openstack controller to create new VMs and add them to the cluster. Conversely, VMs will be removed from the cluster if the average resource utilization of this cluster is below the predefined lower threshold. The implementation of autoscaling is closely related to the monitoring of the VMs, the determination of the autoscaling controller and the VM provision of the Openstack controller. If any component fails, the autoscaling of the cluster will not succeed. The autoscaling process can be described as an event sequence 〈*VM* *monitoring*, *autoscaling* *determinination*, *VM* *provison*〉. The resource utilizations of the VMs are affected by various factors, such as user behavior and other VMs sharing the same physical resources. These factors are time-varying and unpredictable, so we cannot obtain the average resource utilization of the cluster. Thus, we cannot determine the quantity of VMs to add or remove. A test oracle is not attainable in this process, which is an apparent oracle problem. MT can be used to alleviate this problem.

#### Metamorphic relations of autoscaling on an elastic cloud management system

In general, the evaluation of the autoscaling of an elastic cloud management system includes the following two perspectives.

- How accurately are the resources provided according to the workload variation and autoscaling strategy?
- How quickly or timely are the resources provided in an elastic cloud management system or platform?

The following two metrics are considered in this case study as good indicators of autoscaling.

- Scaling resource ability: the ability to scale out or scale in resources to match workload variation.
- Scaling resource time: the response time to scale out or scale in resources.

For any web application cluster in this case study, the upper and lower thresholds of CPU utilization are set to 80% and 20% in its autoscaling strategy, respectively. Each autoscaling has two evaluation periods, and each evaluation period lasts for a certain determination time. The cluster will not scale out a virtual machine until the average resource utilizations in the two evaluation periods both exceed 80%. Moreover, if the average CPU utilizations in two consecutive evaluation periods are both below 20%, the elastic cloud management system will scale in one VM from the web application cluster. A virtual machine can usually be provided within a minute. In fact, the time for scaling out may be delayed due to network speed and disk I/O speed. We suppose that two completely identical web application clusters, including the same resource and running environment, exist. The clusters can scale out or scale in resources according to the same autoscaling strategy. The following MRs can be constructed.

1. **MR1:** If the same workload is imposed on two identical clusters during the same observation period, they will increase by the same number of VMs.
2. **MR2:** In contrast to *MR*1, the two identical clusters will decrease by the same number of VMs when their workloads decrease by the same amount during the same observation period.
3. **MR3:** During the same observation period, if two identical clusters are both stressed with the same workload that causes their CPU utilizations to exceed 80%, they should scale out the same number of VMs, and their response time for scaling out VMs should be similar at the minute level. That is, if one cluster scales out one VM within *t* minutes, then the scaling-out time of the other cluster should be in the range *t* ± 1 minutes.

#### Experimental results and analysis

To test the autoscaling of a cloud management system, we need to simulate the workload of an application to trigger resource autoscaling. The load test software ‘webbench’ is used to impose a workload on a web application. This software can concurrently simulate thousands of requests to visit a web application per second, which can cause the resource utilization of the cluster running this web application to increase sharply. Autoscaling of this cluster can thus be triggered. Note that workload generation is not the event being tested but the method used to generate test cases. We create two groups of clusters with the same resource configuration but different operating systems. One group includes two clusters using the ‘CentOS 6.5 Server’ operating system, and the other group includes two clusters running the ‘Ubuntu 14.04 Desktop’ operating system. Each cluster includes 1 loadbalance (LB) service, 3 Tomcat web servers based on VMs and 1 MySQL database server based on a VM. Each cluster is reset to the initial quantity of VMs before each test is implemented. Furthermore, these VMs are all 2*vCPU*/2*G*/40*G* (2 core CPU, 2 *GB* memory and 40 *GB* disk).

For each MR, we use the ‘webbench’ software to generate workloads as test cases. For example, the identical source and follow-up test cases can be generated by executing the command ‘webbench -c m -t h http://192.168.80.12/’ for *MR*1, where *m* and *h* can be set to random values within the range [3000, 20000] and [0, 3600], respectively. *m* concurrent processes of visiting a web site are executed to generate workloads within *h* seconds. The resource utilization increases sharply to over 80% and then remains above 80%. For *MR*2, we first stress two identical clusters to make their resource utilizations exceed 20% during the same period, and then interrupt the stress operations simultaneously. Thus, their workloads decrease quickly, and these clusters will scale in VMs. The source and follow-up test cases are both generated via the above process. For *MR*3, the source and follow-up test cases can be constructed in the same manner as those of *MR*1. For each MR, we separately construct 100 source test cases and 100 follow-up test cases to test each group of clusters.

Autoscaling of a cluster involves not only the three components of the elastic cloud management system but also the related component of the Openstack cloud platform. The components are developed based on different programming languages and operating systems. Mutation analysis is not suitable for testing this system. We use three different program versions (*V*1.0, *V*2.0 and *V*3.0) to verify the effectiveness of our approach in the development process of the system. Each program version provides the functions of monitoring, autoscaling and resource provisioning. Program *V*1.0 is the first version submitted by the development team. The first version was revised to program *V*2.0 because of some faults. In the new program *V*2.0, the CPU monitoring interval is set to 600 *s*. The autoscaling determination time is set to 600 *s* per evaluation period. In the further revised program *V*3.0, the CPU monitoring interval is set to 300 *s*, and the autoscaling determination time per evaluation period is the same as that in program *V*2.0.

We used the above three MRs to test each program version. All test cases were executed, and their outputs were compared to verify whether they violated these MRs. The experimental results are presented in Table 12. *MR*1 and *MR*2 are not violated with the FDR of 0%, while *MR*3 is violated with the FDR of 100% for program *V*1.0. No cluster adds or removes any VM under the different application workloads. The development team reviewed the program and found that the user of the ceilometer component had no right to access the MongoDB database. Therefore, the corresponding monitoring data were not saved to the MongoDB database, and autoscaling was not triggered. For program *V*2.0, all MRs are violated. The development team found that the cloud management system retrieved insufficient data from the MongoDB database in some cases, which prevented the autoscaling from being triggered to scale out resources. In general, the monitoring data are first saved to the MongoDB database; then, the autoscaling controller retrieves the monitoring data from the database to determine whether to trigger autoscaling. The determination time of autoscaling per evaluation period should be longer than the monitoring interval to obtain sufficient data. This problem of program *V*2.0 is fixed in program *V*3.0. According to the experimental results of program *V*3.0, only *MR*3 is violated. This result demonstrates that resetting the monitoring interval greatly alleviates the problem of insufficient data. However, the response time problem of resource provisioning remains in the autoscaling process. The development team found that the GUIs of the ‘Ubuntu 14.04 Desktop’ operating system on some VMs did not start or started very slowly, which caused a longer time for scaling out resources and violated *MR*3. *MR*3 is more effective than the others due to its richer output relations (i.e., scenarios). Three actual problems are found in the testing process of MTES. One is the image problem from the VM provisioning component of the Openstack cloud platform, and the others are configuration problems from the monitoring component. The results show that MTES is applicable and simple in the domain of cloud computing.

**Table 12:** Fault-detection rates of MRs.

Version of Program	MR1	MR2	MR3
V1.0	0%	0%	100%
V2.0	28%	23%	35%
V3.0	0%	0%	12%

### Summary of the experimental findings

According to the above results and analysis for the case studies, we summarize the experimental findings as follows.

1. MRs based on different event sequences have higher fault-detection capabilities due to more different test scenarios.
2. MRs with richer input and output relations have higher fault-detection capabilities.
3. Different MRs have different sensitivities to different types of mutants. Those with an addition transformation or a permutation transformation in the input sequences have difficulty detecting off-by-one mutants.
4. Good MRs are those that make the source execution and follow-up execution as different as possible. This confirms the findings of two previous studies [41, 42]. Furthermore, the differences in the source and follow-up executions in this paper include different event sequences, different execution paths, different input and output parameters.
5. MRs based on event sequences exhibit high effectiveness, with an MS up to 89% in the fine-grained module testing and 100% for some types of mutants in case study 2. However, only 39.23% of all mutants are killed in the system testing of case study 1. Therefore, MTES is not always efficient but makes it easy for end-users to test systems with rich business processes.

## Discussion

We concluded in our previous work [43] that MT is a cost-effective approach for factual applications with mathematical functions. In this paper, we propose an approach to construct MRs between event sequences, which can construct multiple types of metamorphic relations to test various business processes of actual applications. The effectiveness and applicability of the proposed approach are validated via case studies.

### More general application in different domains

In the IT industry, an increasing number of applications integrate several systems or services to provide business processes. Particularly, many cloud applications integrate a large number of cloud services and involve various scenarios of business processes; therefore, the oracle problem has become a critical issue. In reality, users pay more attention to the correctness of business processes. However, previous studies have seldom employed MT techniques to test various business processes. Our approach applies MT to test business-process-based software systems, and its applicability and effectiveness are verified through three case studies in different domains. The proposed method is a general approach that can be used in applications from other domains.

Additionally, our approach introduces the process of MTES to verify the correctness of business processes. The MT is refined in terms of the identification of business processes and the construction of MRs. In MTES, business process scenarios are first identified based on the domain knowledge of experts or users. Then, the corresponding event sequences are organized to construct MRs. These are the general components of MTES that are suitable for applications in different domains.

More importantly, we not only use the rules from the previous studies to construct MRs between event sequences but also extend the guidance on the construction of good MRs. Good MRs should not only make the executions of the SUT as different as possible but also make the input and output relations as rich as possible. Moreover, the differences in executions should also include different event sequences and different input and output parameters, with the exception of different execution paths.

MTES can not only alleviate the oracle problem in business process testing but also make the testing of some business processes easier and more efficient. Generally, business processes with test oracles have simple mathematical or logical relations that must be tested with a large quantity of test data. MTES can use simple relations without the manual and error-prone computations for testing, which is simpler and more efficient than regular testing. The future combination of MTES with automated techniques will further improve the test efficiency of MTES and promote its wide application in the IT industry.

### Limitations

MTES is promising for testing business-process-based software systems. Our approach has certain limitations in the construction of MRs. If more events are involved, MRs between event sequences become more difficult to construct. If a user’s business processes are 4, 5,…,*n*-way event sequences, the number of input and output relations will increase substantially in MRs. Furthermore, a large number of source and follow-up test cases based on these event sequences and MRs will be difficult to generate. The cost will be very high for the implementation of MTES. We could alternatively design simple MRs (e.g., non-equalities) to verify the correctness of business processes to reduce the cost of MTES. However, in this paper, our approach does not focus on the cost of MTES but rather on its feasibility and effectiveness in software systems from different domains. Therefore, we design various MRs with respect to different business processes, different execution paths, and different input and output relations to validate the approach. Although these MRs exhibit higher fault-detection capabilities than those with single-event scenarios, they are relatively complex and difficult to construct. In the future, how much will the MRs and test cases increase when going from 1 event to 2,3,…,*n*-way event sequences? Moreover, what is the most suitable dimension for an event sequence to balance the effectiveness and cost of MTES? These problems require additional research.

### Validity

The primary threat to internal validity is the implementation of MTES, such as test case generation, test execution and comparison of test outputs. We tested the implementation at the unit level and system level and checked the data thoroughly. We also adopted measures to resolve the problems related to floating-point precision and rounding when test outputs are compared. These steps ensured the quality of our experiments.

The threat to external validity is mainly related to the systems under test. In this paper, the system under test in case study 2 was used in our previous study [43]. The system in case study 1 is similar to that in case study 2. They are the simplified programs with mathematical functions from real-life applications. Although these systems are small, they have common characteristics with business process scenarios. The system in case study 3 is a real-life elastic cloud management system involving a complex cloud resource environment and event relations. The oracle problem is prominent. These three systems from different domains are typical and meaningful to expand the application of MTES in the software industry. It is also worthwhile to further investigate the effectiveness of our approach with respect to other classes of systems in the software industry.

Another threat to external validity is the mutants automatically generated by the mujava tool in case study 1 and 2. Although the mutants generated by mutation operators are similar to real faults [38], they are not real faults and can be restricted in type. However, mutation analysis has been widely used to evaluate the effectiveness of test methods, so this threat is acceptable. In addition, we use three different program versions with real faults in case study 3 to validate our approach. The experimental results are also promising.

The primary threat to construct validity is the measurement of the effectiveness. We use the MS and fault-detection rate as metrics of the effectiveness of the MRs. These metrics have been widely used in the literatures. Another threat to construct validity is the construction of MRs for event sequences. Because MRs for event sequences involve various business processes of different systems from multiple domains, we may not be fully acquainted with them. Experts from these domains gave us professional guidance to ensure the correctness of the MRs constructed for event sequences, thereby greatly reducing the threat.

## Related work

Some researchers have applied MT to system testing and integration testing. Murphy et al. proposed an automatic system testing approach and its implementation framework [8]. Their study focused on the automation of MT, such as automatic input transformations, parallel executions and output comparisons of applications. However, our approach focuses on the construction of MRs between event sequences. Chan et al. proposed the concept of checkpoints, which provided a convenient way to conduct integration testing of middleware-based applications [44]. They used the relations of the source and follow-up input sequences between checkpoints to test the program, which is, to some extent, similar to our approach. However, our approach includes not only the relations between the source and follow-up input sequences but also the relations between the source and follow-up event sequences. Our approach is more specific and feasible for practical applications.

Some researchers have applied MT in the domain of bank and cloud computing. Chan et al. proposed a metamorphic approach for online service testing and conducted a case study on a foreign exchange dealing service applications [45]. They used the successful test cases of offline testing as the source test cases for online testing, but they assumed that test oracles were available for offline testing. Our method does not include this assumption. Sun et al. proposed an MT framework for web services and conducted a case study on a transfer function of a bank system [46]. However, they designed only simple MRs, most of which were non-equalities. In this paper, we consider different business process scenarios to design different types of MRs to demonstrate that MT is suitable and effective for systems with various business process scenarios. A methodology is proposed to semi-automatically test and validate cloud models by combining simulation techniques and MT [47]. The method simulates different cloud models and constructs different MRs to implement performance experiments, which validate the usefulness and applicability of MT in cloud computing. In contrast to this study, our approach focuses on function testing of a cloud management platform. We provide an effective approach to constructing MRs between event sequences to test business processes, which can easily be extended to test applications from different domains.

To some extent, we reference to event sequence generation and test case generation from GUI testing [25, 26, 28, 48], but we further integrate these generation methods with MT and propose MTES to test business-process-based software systems. Moreover, these GUI testing methods regard only direct-interactive events as an event sequence, whereas we also regard related events as an event sequence.

Additionally, some researchers have proposed principles for constructing good MRs. Murphy et al. [49] suggested input transformation rules to construct MRs for mathematical functions, such as permutation, addition and multiplication. Chen et al. [50] proposed a METRIC identification methodology based on the category-choice framework and developed a generator tool, MR-GEN, to help users identify MRs from specifications in a systematic manner. This methodology improved the applicability, effectiveness and automation of MT. Mayer and Guderlei [51] derived that some MRs with linear equations, as well as those close to the implementations, are limited in terms of fault-detection capability. They proposed that good MRs should have rich semantics. Sun et al. proposed an acquisition methodology (*μ*MT) of MRs by means of data mutation [52], in which data mutation operators are applied to generate valid mutated test cases as follow-up test cases and the output relations are generated according to the input relations by the mapping rules. Ding and Zhang proposed an approach to iteratively refine MRs for adequate tests [53]. This approach first constructs initial MRs to implement mutation testing and then evaluates the effectiveness of metamorphic relations to iteratively refine MRs. Liu et al. [54] proposed a composite approach of MRs to achieve higher cost-effectiveness with respect to an event, algorithm or function. Although these approaches indicated how to construct good MRs, they did not provide guidance for the construction of MRs for event sequences. This paper proposes some general rules, called properties between event sequences, to construct MRs for business processes.

## Conclusion

Many studies have demonstrated that MT is an effective approach to test programs with test oracle problems. However, most of these studies have not considered rich business process scenarios in the software industry. Therefore, the applicability of MT requires further validation. In this paper, we propose an MT approach for event sequences, which can be used to systematically test applications with rich business processes. We conduct three case studies in different domains to illustrate our approach. The experimental results demonstrate the feasibility and effectiveness of our approach. The results also confirm the previous findings that good MRs are those that make the executions as different as possible. Furthermore, this paper considers more differences between the source and follow-up executions, such as different event sequences and different input and output parameters and relations. We find that MRs based on different event sequences have higher fault-detection capabilities than those based on the same event sequence. Additionally, MRs with richer input and output relations have higher fault-detection capabilities. On the other hand, to improve the practical impact of our proposed approach, more experimental studies involving real-world software applications and applications suffering from the oracle problem should be conducted. This will be an important aspect of our future work.
